# Identifying potential monkeypox virus inhibitors: an *in silico* study targeting the A42R protein

**DOI:** 10.3389/fcimb.2024.1351737

**Published:** 2024-03-04

**Authors:** Carolyn N. Ashley, Emmanuel Broni, Chanyah M. Wood, Tunmise Okuneye, Mary-Pearl T. Ojukwu, Qunfeng Dong, Carla Gallagher, Whelton A. Miller

**Affiliations:** ^1^ Department of Medicine, Loyola University Medical Center, Loyola University Chicago, Maywood, IL, United States; ^2^ Department of Chemistry and Physics, Lincoln University, Lincoln, PA, United States; ^3^ Department of Biology, Lincoln University, Lincoln, PA, United States; ^4^ College of Pharmacy, University of Florida, Orlando, FL, United States; ^5^ Center for Biomedical Informatics, Stritch School of Medicine, Loyola University Chicago, Maywood, IL, United States; ^6^ Department of Molecular Pharmacology & Neuroscience, Loyola University Medical Center, Loyola University Chicago, Maywood, IL, United States

**Keywords:** monkeypox virus, orthopoxviruses, tecovirimat, molecular docking, molecular dynamics simulation, ADMET, biological activity prediction

## Abstract

Monkeypox (now Mpox), a zoonotic disease caused by the monkeypox virus (MPXV) is an emerging threat to global health. In the time span of only six months, from May to October 2022, the number of MPXV cases breached 80,000 and many of the outbreaks occurred in locations that had never previously reported MPXV. Currently there are no FDA-approved MPXV-specific vaccines or treatments, therefore, finding drugs to combat MPXV is of utmost importance. The A42R profilin-like protein of the MPXV is involved in cell development and motility making it a critical drug target. A42R protein is highly conserved across orthopoxviruses, thus A42R inhibitors may work for other family members. This study sought to identify potential A42R inhibitors for MPXV treatment using computational approaches. The energy minimized 3D structure of the A42R profilin-like protein (PDB ID: 4QWO) underwent virtual screening using a library of 36,366 compounds from Traditional Chinese Medicine (TCM), AfroDb, and PubChem databases as well as known inhibitor tecovirimat via AutoDock Vina. A total of seven compounds comprising PubChem CID: 11371962, ZINC000000899909, ZINC000001632866, ZINC000015151344, ZINC000013378519, ZINC000000086470, and ZINC000095486204, predicted to have favorable binding were shortlisted. Molecular docking suggested that all seven proposed compounds have higher binding affinities to A42R (–7.2 to –8.3 kcal/mol) than tecovirimat (–6.7 kcal/mol). This was corroborated by MM/PBSA calculations, with tecovirimat demonstrating the highest binding free energy of –68.694 kJ/mol (lowest binding affinity) compared to the seven shortlisted compounds that ranged from –73.252 to –97.140 kJ/mol. Furthermore, the 7 compounds in complex with A42R demonstrated higher stability than the A42R-tecovirimat complex when subjected to 100 ns molecular dynamics simulations. The protein-ligand interaction maps generated using LigPlot+ suggested that residues Met1, Glu3, Trp4, Ile7, Arg127, Val128, Thr131, and Asn133 are important for binding. These seven compounds were adequately profiled to be potential antivirals via PASS predictions and structural similarity searches. All seven potential lead compounds were scored Pa > Pi for antiviral activity while ZINC000001632866 and ZINC000015151344 were predicted as poxvirus inhibitors with Pa values of 0.315 and 0.215, and Pi values of 0.052 and 0.136, respectively. Further experimental validations of the identified lead compounds are required to corroborate their predicted activity. These seven identified compounds represent solid footing for development of antivirals against MPXV and other orthopoxviruses.

## 1 Introduction

Mpox is a zoonotic disease caused by infection from the monkeypox virus (MPXV) (Tayyaba et al., 2022; Kandra et al., 2023). Two genetically distinct clades have been identified i.e., the Congo basin (Central African) clade and the West African clade, with the Congo clade being more frequently reported, more virulent, and having more documentation of human transmission (Kabuga and El Zowalaty, 2019; Sadeuh-Mba et al., 2019; Zardi and Chello, 2022). In 2022, an unusual wave of MPXV resurfaced with cases identified in over 100 non-endemic countries or regions and has increased the possibility of another global health crisis (Zardi and Chello, 2022).

MPXV can be transmitted from person to person through close contact or by encountering bodily fluids or sores of an infected person or animal (Zardi and Chello, 2022). Zoonotic transmission of MPXV occurs through direct contact with or consumption of animal hosts including non-human primates, but more commonly including rodents such as tree squirrels, Gambian pouched rats, and dormice (Kabuga and El Zowalaty, 2019; Zardi and Chello, 2022; Li et al., 2023). Early MPXV symptoms begin with a fever followed by an evolving rash characterized by different skin lesions and a swelling of the lymph nodes which distinguishes MPXV from other orthopoxviruses such as smallpox caused by the variola virus (VARV) (Kandra et al., 2023). The number of lesions can be severe and affect sensitive areas such as the genitals or oropharynx that can make MPXV extremely painful (Hallo-Carrasco et al., 2023). Significant amounts of lesions on the genitals can often cause misdiagnosis of MPXV as syphilis or other sexually transmitted infections (Cohen, 2022). A large portion of MPXV patients also suffer from human immunodeficiency virus (HIV), as a report by the Centers for Disease Control and Prevention (CDC) shows that 82% of 57 patients (≥18 years) hospitalized between August 10 to October 10, 2022, were co-infected with HIV (Miller et al., 2022). Reports of PCR results from semen samples positive for MPXV suggest the possibility of MPXV being spread via sexual transmission (Antinori et al., 2022).

MPXV has an incubation period anywhere from 3 to 17 days and a full recovery without significant complications can normally span from 2 weeks to a month. While infected, patients require hospitalization and single room isolation to control spread of infection and for pain management (Hallo-Carrasco et al., 2023). MPXV can be fatal or cause severe complications including pneumonia, sepsis, encephalitis, and loss of vision as a result of eye infections (Huang et al., 2022). Fatality rates in African countries have had at least 75 confirmed deaths (Tomori and Ogoina, 2022). In non-endemic countries from August 10^th^ to October 10^th^ the CDC reported that 30% of patients required ICU-level care and there were 12 deaths, 5 of which MPXV was the cause or major contributing factor of fatality (Miller et al., 2022). Stay in the intensive care unit (ICU) and prolonged hospitalization while immunocompromised can also increase risk of secondary illnesses by nosocomial infections (de la Calle-Prieto et al., 2023; da Costa et al., 2022; Jeon et al., 2012). Those most at risk for acquiring MPXV are usually children, those in contact with animal hosts, and patients suffering from other conditions (Huang et al., 2022).

MPXV was first identified in 1958 as a pox-like disease outbreak in monkeys kept at a research institute in Copenhagen, Denmark (Parker and Buller, 2013). The first human case of MPXV occurred in 1970 in a nine-month-old boy in the Democratic Republic of Congo (Meo and Ali Jawaid, 2022; Bunge et al., 2022). It took until 2003 for the first case of MPXV outside of Africa to be reported in the United States (Antinori et al., 2022; Meo and Ali Jawaid, 2022). Since then, there have been few other sporadic cases up until 2017 and especially 2022 (Zardi and Chello, 2022). Starting in May of 2022 there was a rapid increase in MPXV cases with outbreak of MPXV in over 100 locations with no prior reported cases (Zardi and Chello, 2022). In the time span from May to October 2022 the CDC reported 86,500 global cases of MPXV with 30,262 being in the United States (Owens et al., 2023). Even with the significant increase in MPXV cases, there are very few treatment options available, and no FDA-approved drugs that are MPXV specific.

There are some preventative measures for MPXV through vaccination using FDA approved vaccines ACAM2000 or JYNNEOS. ACAM2000 is reported to have good efficacy but has issues with negative side effects including myopericarditis (Zardi and Chello, 2022). JYNNEOS requires further testing to validate its efficacy (Xiang and White, 2022). Both vaccines have been licensed for the treatment of smallpox and have been shown to lower the risk of MPXV infection (Zardi and Chello, 2022). Drug treatments available for MPXV include tecovirimat and brincidofovir (Niaz et al., 2022). Both drugs target smallpox, but due to genetic similarity among poxviruses, they show promise in MPXV at least in animal models (Adler et al., 2022; Frenois-Veyrat et al., 2022; Sherwat et al., 2022; Warner et al., 2022). Tecovirimat targets a highly conserved protein in poxviruses, p37, and brincidofovir targets DNA replication of the poxviruses (Xiang and White, 2022). Notably, brincidofovir has had complications of toxicity when used in human patients (Adler et al., 2022). Tecovirimat can successfully decrease viral shedding and length of illness, but there is minimal data on human efficacy of tecovirimat for MPXV (Adler et al., 2022; Sherwat et al., 2022). Vaccines are limited in quantity and accessibility and treatment options are limited for MPXV; therefore, it is of utmost importance to search for new antivirals to combat MPXV (Chakraborty et al., 2022).

MPXV are large enveloped double stranded DNA viruses around 200-250 nm and identifiable by their brick-shape, surface tubules, and dumbbell-shaped core (Parker and Buller, 2013). MPXV and other poxviruses avoid the host cell’s nucleus and carry out replication and viral assembly in the cytoplasm (Realegeno et al., 2017). In poxviruses, the mature virions (MV) are trapped in the intracellular space of a host cell unless transported to the golgi for extra preparation and wrapping to exit the cell as an extracellular virion (EV) (Realegeno et al., 2017). The EV form of MPXV is necessary for cell motility and viral spread through the host (Realegeno et al., 2017). To get to the EV form, the MV must traverse to the plasma membrane of the host cell using microtubules, and once the virus is coated in a membrane it can fuse into the cell membrane and exit the cell via different mechanisms (Duncan et al., 2018; Meiser et al., 2003). One mechanism is the production of an actin tail used for motility of the EV to a neighboring cell (Zhang et al., 2000). Actin tail production has been shown in other poxviruses to be a critical factor in viral release from an infected cell (Duncan et al., 2018).

In this study MPXV A42R profilin-like protein was used as the drug target. Profilins are important in cell motility by interacting with actin and influencing cytoskeletal dynamics (Butler-Cole et al., 2007) although A42R only weakly interacts with actin unlike other cellular profilins (Minasov et al., 2022). Actin is important in the pathogenicity of other poxviruses by impacting viral spread to neighboring cells. In a study of another profilin homolog of a different poxvirus, ectromelia virus, it was observed that alpha-tropomyosin directly interacts with the viral profilin-like protein (Butler-Cole et al., 2007). Using immunofluorescence, it was suggested that alpha-tropomyosin may colocalize with actin-tail-like structures or surface tubules (Butler-Cole et al., 2007). This colocalization suggests that tropomyosin is involved in the motility of the virus. A42R also interacts with phosphatidylinositol lipids (Minasov et al., 2022). Viral interactions with lipids are a mechanism used to alter the host cell and support the wrapping of the virus needed for efficient fusion to the plasma membrane and then protection in the cytoplasm (Heaton and Randall, 2011). Profilin-like proteins in vaccinia virus (VACV) interact more strongly with the polyphosphatidylinositides (PPI) than actin (Machesky et al., 1994). A42R in a structural comparison to cellular profilins suggest that MPXV may also bind PPIs with a higher affinity than actin (Minasov et al., 2022). Therefore, A42R is likely more related to the regulation of phosphatidylinositol metabolism rather than actin structure, but important in membrane trafficking and cell motility. When looking at sequence alignments of A42R across orthopoxviruses, it is a highly conserved protein, with its most distant homolog being 79% identical (Minasov et al., 2022). These roles in viral infection and the conservation of A42R in other relatives support its role as a critical therapeutic target for MPXV.

Due to the high cost and time inefficiencies of traditional drug development, computer-aided drug design (CADD) methods are receiving a lot of recognition regarding identifying therapeutics that are specific and selective against viral pathogens. The identification of new compounds can be screened by combined applications of CADD to help the development of future antiviral drugs. Therefore, the identification of new bioactive compounds via *in silico* drug design is vital in the discovery of new leads that have the potential to inhibit A42R. This study therefore sought to identify potential therapeutic candidates through virtual screening and to characterize the binding mechanisms between the A42R and potential inhibitory molecules by utilizing molecular dynamics (MDs) simulations and molecular mechanics Poisson-Boltzmann surface area (MM/PBSA) methods.

## 2 Results

### 2.1 Protein structure and binding site prediction

The A42R protein retrieved from the Research Collaboratory for Structural Bioinformatics Protein Data Bank (RCSB PDB) had two chains, A and B. Each chain is composed of seven antiparallel β-sheets, three α-helices and a partial helix (Minasov et al., 2022). Herein, the A42R structure was subjected to energy minimization using all-atom optimized potentials for liquid simulations (OPLS/AA) and Chemistry at Harvard Macromolecular Mechanics 36 (CHARMM36) force fields. The A42R protein structure was minimized using the OPLS/AA force field that had a lower potential energy of –3.896 × 10^5^ kJ/mol in 667 steps than that of CHARMM36, which converged in 374 steps with an energy of –3.709 × 10^5^ kJ/mol (**Supplementary Figure S1**). Thus, the A42R structure which was energy minimized using OPLS was used in this study due to its lower energy which implies a higher stability (Pallio et al., 2023).

Computed Atlas of Surface Topology of proteins (CASTp) 3.0’s (Tian et al., 2018) prediction of binding pockets for A42R resulted in pocket 1 as the largest with an area of 115.519 Å^2^ and a total volume of 31.983 Å^3^ (**Table 1**). Upon visualizing the other pockets in PyMOL, it was observed that the other pockets were relatively small and could not accommodate ligands. Residues lining pocket 1 included Met1, Glu3, Trp4, Lys6, Ile7, Asp10, Ile22, Thr99, Ile104, His124, Ala125, Arg127, Val128, Thr131, and Asn133 (**Table 1**). Of the twenty predicted binding pockets, pocket 1 stood out as the largest cavity and was the only binding site of A42R assessed in this study.

**Table 1:** Four largest predicted binding cavities via CASTp with their area, volumes and residues lining each pocket.

Pocket No.	Area (Å²)	Volume (Å^3^)	Residues lining the Pocket
1	115.519	31.983	Met1, Glu3, Trp4, Lys6, Ile7, Asp10, Ile22, Thr99, Ile104, His124, Ala125, Arg127, Val128, Thr131, and Asn133.
2	37.568	17.259	Glu18, Thr86, Tyr88, Ala89, Pro90, Ser92, Met107, Lys109, and Pro110.
3	19.000	2.456	Ile7, Ile8, Ile11, Ala20, Ala21, Ile22, Ile104, Leu106, Cys121, and His124.
4	11.978	1.143	Leu51, Ile52, Thr53, Asn54, His55, Asn72, and Met75.

### 2.2 Molecular docking via AutoDock Vina

AutoDock Vina module in PyRx version 0.9.2 successfully screened 26,315 compounds (25,196, 821, and 298 from TCM, AfroDb, and PubChem, respectively) against MPXV A42R profilin-like protein (Trott and Olson, 2010; Dallakyan and Olson, 2015). Docking conformations were visualized for compounds with the lowest docking scores (highest binding affinities). Compounds were checked for binding to pocket 1, the most plausible binding site. TCM compounds ZINC000070455208 and ZINC000085543530, both with the lowest docking score at –9.0 kcal/mol, and ZINC000043552595 at –8.9 kcal/mol were eliminated as they did not bind to pocket 1. Similarly, ZINC000095485942 from AfroDb with a docking score of –8.5 kcal/mol was eliminated. After eliminating compounds not bound to pocket 1, the pose with the most negative docking score was selected as the best for each ligand. A previous docking study screening *Plantago lanceolate* compounds against A42R resulted in comparable binding energies ranging from –5.3 to –9.9 kcal/mol (Bajrai et al., 2022). The top 1% from TCM was shortlisted comprising 252 compounds with docking scores of –7.7 kcal/mol or less. All compounds passing below the –7.0 kcal/mol threshold were retained from AfroDb and PubChem leaving 44 and 3 respectively. Other molecular docking studies screening for compounds against A42R resulted in binding energies greater than –6.8 kcal/mol (Burkhanova et al., 2022; Preet et al., 2022). The lowest docking score from TCM was observed for ZINC000043552595 with –8.8 kcal/mol, from AfroDb was ZINC000095486204 with –8.3 kcal/mol, and from PubChem CID: 11371962 with –7.2 kcal/mol. These are considered good especially in comparison to tecovirimat, a known MPXV inhibitor whose binding energy was above the –7.0 kcal/mol threshold at –6.7 kcal/mol. It is worth noting that tecovirimat has not been shown to target the A42R protein. Other top compounds included ZINC000000899909, ZINC000001632866, ZINC000015151344, ZINC000013378519, and ZINC000000086470. Interactions such as hydrophobic and hydrogen bonds are important for the stability of the ligand binding to the A42R interface (**Table 2**). Optimization of both hydrophobic and hydrogen bonds can be used to improve drug selectivity to reduce off-target adverse effects and improve efficacy (Patil et al., 2010).

**Table 2:** Binding energies from AutoDock Vina of the seven lead compounds and tecovirimat with A42R.

Compound	Binding Energy (kcal/mol)	Interacting Residues	
		Hydrophobic Bonds	Hydrogen Bond (Bond Length, Å)
Tecovirimat	-6.7	Glu3, Trp4, Ile7, Arg127, Val128, Thr131, Asn133	Met1 (3.19 and 3.33)
PubChem CID: 11371962	-7.2	Glu3, Trp4, Ile7, Arg127, Val128, Thr131, Asn133	Met1 (3.08)
ZINC000000899909	-7.8	Glu3, Trp4, Ile7, Arg127, Val128, Thr131, Asn133	Met1 (3.19)
ZINC000001632866	-8.0	Met1, Glu3, Trp4, Ile7, Arg127, Val128, Thr131, Asn133	–
ZINC000015151344	-7.9	Glu3, Trp4, Arg127, Val128, Thr131	Met1 (3.16) and Asn133 (3.29)
ZINC000013378519	-8.1	Met1, Glu3, Trp4, Lys6, Ile7, Asp10, Arg127, Val128, Thr131, Asn133	–
ZINC000000086470	-7.6	Glu3, Trp4, Ile7, Arg127, Val128, Thr131, Asn133	Met1 (3.16) and Met1 (2.79)
ZINC000095486204	-8.3	Glu3, Trp4, Arg127, Val128, Thr131, Asn133	Met1 (3.17)

The interacting residues and the type of interaction is presented for each protein-ligand complex.

### 2.3 Molecular interactions between A42R and top compounds

**Figure 1:**
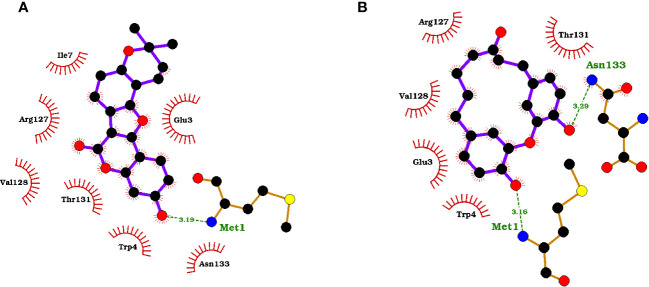
Protein-ligand interaction maps of two top compounds presenting important binding residues for compounds **(A)** ZINC000000899909 and **(B)** ZINC000015151344. For the interaction maps, black circles are carbon, red circles are oxygen, blue circles are nitrogen, and yellow circles are sulfur. Residue names in green interact in hydrogen bonding, dashed green lines are the hydrogen bond representation. Black residues are interacting by hydrophobic bonds corresponding to red markings on the ligands.

The protein-ligand interaction maps for the seven potential candidates and tecovirimat are presented in (**Figure 1**; **Supplementary Figure S2**). Tecovirimat (PubChem CID: 16124688) interacted with A42R residues Glu3, Trp4, Ile7, Arg127, Val128, Thr131, and Asn133 via hydrophobic bonds and formed two interactions with Met1 via hydrogen bonding with lengths 3.19 and 3.33 Å (**Supplementary Figure S2F**). PubChem CID: 11371962 interacted with A42R residues Glu3, Trp4, Ile7, Arg127, Val128, Thr131, and Asn133 via hydrophobic bonds and one 3.08 Å hydrogen bond with Met1 (**Supplementary Figure S2A**). ZINC000000899909 formed hydrophobic bonds with residues Glu3, Trp4, Ile7, Arg127, Val128, Thr131, and Asn133 and a hydrogen bond with Met1 (3.19 Å) (**Figure 1A**). ZINC000001632866 formed hydrophobic bonds with residues Met1, Glu3, Trp4, Ile7, Arg127, Val128, Thr131, and Asn133 (**Supplementary Figure S2B**). ZINC000015151344 formed hydrophobic bonds with residues Glu3, Trp4, Arg127, Val128, and Thr131 and two hydrogen bonds with residues Met1 and Asn133 with bond lengths 3.16 and 3.29 Å, respectively (**Figure 1B**). ZINC000013378519 formed hydrophobic bonds with residues Met1, Glu3, Trp4, Lys6, Ile7, Asp10, Arg127, Val128, Thr131, and Asn133 (**Supplementary Figure S2C**). ZINC000000086470 formed hydrophobic bonds with residues Glu3, Trp4, Ile7, Arg127, Val128, Thr131, and Asn133, as well as two hydrogen bonds with Met1 of bond lengths 3.16 and 2.79 Å (**Supplementary Figure S2D**). ZINC000095486204 formed hydrophobic bonds with residues Glu3, Trp4, Arg127, Val128, Thr131, and Asn133 and a hydrogen bond with Met1 of bond length 3.17 Å (**Supplementary Figure S2E**). In all 8 compounds, the residues Met1, Glu3, Trp4, Arg127, Val128, Thr131, and Asn133 were involved in protein-ligand interactions. Met1 was involved in at least one hydrogen bond for 6 of the 8 compounds and involved in 2 hydrogen bonds for tecovirimat and ZINC000000086470. Ile7 was also a prevalent interaction residue involved in 6 of the 8 protein-ligand interactions. Other studies that looked at interacting residues between A42R and ligands have also mentioned Trp4 and Arg127 involvement (Minasov et al., 2022; Dassanayake et al., 2022).

### 2.4 ADMET prediction

There were a total of 111 compounds that failed ADME and were eliminated from consideration. These included compounds that violated more than one of Lipinski’s rules or any violations to Veber’s rule. The top compound ZINC000043552595 violated Veber’s rule and was eliminated (**Table 3**). Twenty other compounds with low docking scores from TCM failed ADME leaving the top compound from TCM to be ZINC000013378519 with a docking score of –8.1 kcal/mol. Top compounds from AfroDb and PubChem remained as ZINC000095486204 at –8.3 kcal/mol, and PubChem CID: 11371962 at –7.2 kcal/mol. A total of 142 out of 252 passed, 43 out of 44 passed, and 3 of 3 passed for TCM, AfroDb, and PubChem, respectively. The shortlisted compounds had a molecular weight between 242.31 g/mol and 482.52 g/mol and TPSA’s ranging up to 88.38 Å^2^ (**Table 3**).

**Table 3:** Pharmacokinetic evaluation of the 8 potential lead compounds and Tecovirimat. The consensus logP value (SwissADME) is reported in this table.

Compound	MW (g/mol)	logP o/w	TPSA (Å²)	BBB Permeant	GI Ab-sorption	ESOL Solubility Class	No. of Lipinski’s rule violations	No. of Veber’s rule violations
Tecovirimat	376.33	2.76	66.48	Yes	High	Soluble	0	0
PubChem CID: 11371962	364.32	2.79	66.48	Yes	High	Soluble	0	0
ZINC000043552595	552.48	5.44	170.8	No	Low	Poor	1	1
ZINC000000899909	336.34	3.81	72.81	Yes	High	Moderate	0	0
ZINC000001632866	242.31	5.26	0	No	Low	Moderate	1	0
ZINC000015151344	312.36	3.29	66.76	Yes	High	Moderate	0	0
ZINC000013378519	482.52	5.19	88.38	No	High	Poor	0	0
ZINC000000086470	336.38	3.69	44.76	Yes	High	Moderate	0	0
ZINC000095486204	363.45	3.9	61.72	Yes	High	Moderate	0	0

BBB permeability should be considered in the adaptation of these compounds as potential MPXV inhibitors. There has been a rising concern about neurological complications associated with MPXV (Sepehrinezhad et al., 2023; Pastula et al., 2022; Billioux et al., 2022). Known neurological symptoms of MPXV have commonly included headache, neuropathic pain, depression, and anxiety (Billioux et al., 2022). A rarer symptom associated with MPXV is encephalitis, but it may be linked to a relatively common symptom, conjunctivitis, that occurs in about 30% of unvaccinated patients (Urmi et al., 2023). In the 2022 outbreak, in an examination of two MPXV patients suffering from encephalitis MPXV DNA was detected in the cerebrospinal fluid (Billioux et al., 2022). It has been reported in MPXV and in other viruses that some ocular symptoms like conjunctivitis may play a role in viral infiltration to the brain resulting in encephalitis (Urmi et al., 2023; Koyuncu et al., 2013; Yue et al., 2022). To address the concern of MPXV populating the brain, the permeability of the compounds to cross the blood brain barrier (BBB) was predicted. From the top compounds PubChem CID: 11371962, ZINC000000899909, ZINC000015151344, ZINC000000086470, and ZINC000095486204 were predicted to be permeable to the BBB (**Table 3**). Excluding the eliminated ZINC000043552595, compounds ZINC000001632866 and ZINC000013378519 were predicted to not cross the BBB, but alternative administration routes could be employed to bypass the BBB (Hersh et al., 2016; Gernert and Feja, 2020; Broni et al., 2023).

Of the twenty-five shortlisted compounds that had high binding affinities to pocket 1 and passed ADME, there was a total of 18 that passed toxicity screening (**Supplementary Table S1**). There were 21, 19, 14, and 23 compounds predicted to have no toxic effects regarding mutagenicity, tumorigenicity, reproductive effects, or irritancy (**Supplementary Table S1**) after subjecting them to toxicity risk prediction using DataWarrior 5.5.0. Ten compounds including ZINC000013378519, ZINC000015151344, ZINC000095909830, ZINC000095913878, ZINC000000689683, ZINC000000897930, ZINC000000134782, ZINC000048998695, ZINC000014557836, and ZINC000038658035 had no predicted toxicity in any of the four categories (**Supplementary Table S1**). Only compound ZINC000095485910 of the top twenty-five had high mutagenic risk. Six compounds had high tumorigenic risk including compounds ZINC000095486204, ZINC000001632866, ZINC000028702248, ZINC000031852149, ZINC000095485910, and ZINC000095486327.

Top compounds ZINC000095486204 and ZINC000001632866 from AfroDb and TCM respectively failed toxicity screening. ZINC000095486204 had both high risk in mutagenicity and low reproductive effect risks while ZINC000001632866 had both low mutagenic and high tumorigenic risks. While this should eliminate them from further use their structures were of interest as they had low docking scores when screened against A42R and good predicted antiviral activity. ZINC000001632866’s binding to A42R was specifically of interest because it had predicted antiviral activity to poxviruses. So, while these drugs should be cautioned against because of their potential toxicity, their structures may be of value in designing new antipoxvirus drugs.

### 2.5 Prediction of biological activities of shortlisted compounds

For each of the seven potential lead compounds PubChem CID: 11371962 (N-(3,5-dioxo-4-azatricyclo[5.2.2.02,6]undec-8-en-4-yl)-4-(trifluoromethyl)benzamide), ZINC000000899909 (Sojagol), ZINC000000086470 (Obovatin 5-Methyl Ether), ZINC000001632866 (3-Methylbenzo[c]phenanthrene), ZINC000095486204 ((1S,3R)-7-(4-hydroxy-5-methoxy-7-methylnaphthalen-1-yl)-1,3-dimethyl-3,4-dihydro-2H-isoquinolin-1-ol), ZINC000013378519 (1-[(7-Hydroxy-4-methoxy-9,10-dihydrophenanthren-2-yl)oxy]-4-methoxy-9,10-dihydrophenanthrene-2,7-diol), and ZINC000015151344 (4,18-Dihydroxy-2-oxatricyclo[13.3.1.13,7]icosa-1(18),3,5,7(20),15(19),16-hexaen-10-one) the probability of activity (Pa) obtained for each activity related to viral inhibition, or antiviral activity was greater than the corresponding probability of inactivity (Pi) using the Prediction of Activity Spectra of Substances (PASS) (Lagunin et al., 2000; Parasuraman, 2011). ZINC000000899909, ZINC000000086470, ZINC000001632866, ZINC000095486204, ZINC000013378519, and ZINC000015151344 were all predicted to inhibit viral entry (**Supplementary Table S2**).

Two compounds that stood out were ZINC000001632866 and ZINC000015151344 which were predicted as antivirals for poxviruses with Pa values of 0.315 and 0.215, and Pi values of 0.052 and 0.136, respectively. These two compounds were also predicted to have antiviral activity to other double stranded DNA viruses in the *Adenoviridae*, *Herpesviridae*, and *Hepadnaviridae* families (**Supplementary Tables S2B, C**). Predicted activity to inhibit adenoviruses had Pa values of 0.387 and 0.381 and Pi values 0.035 and 0.037 for ZINC000001632866 and ZINC000015151344, respectively. In total, each of these two compounds had predicted antiviral activity for ten different viruses, nine of which they had in common including poxviruses, picornavirus, adenovirus, cytomegalovirus (CMV), influenza, herpes, hepatitis C (HCV), rhinovirus, and HIV (**Supplementary Tables S2B, C**). Additionally, ZINC000001632866 was also predicted to have antiviral activity against parainfluenza, and ZINC000015151344 had predicted antiviral activity against hepatitis B (**Supplementary Tables S2B, C**).

MPXV infections can be complicated by other comorbidities. ZINC000000899909, ZINC000001632866, ZINC000015151344, and ZINC000013378519 all had predicted activity to inhibit HIV in several ways by targeting HIV fusion, integration, or reverse transcription and since HIV is a common comorbidity of MPXV these compounds may be useful in further protection from HIV related exacerbations (**Supplementary Tables S2A-D**) (Hoffmann et al., 2022). Human MPXV patients have been reported with inflammation in the spleen and liver though there is a lack of evidence for large amounts of MPXV replication in hepatocytes (Lum et al., 2022). ZINC000000899909, ZINC000013378519, ZINC000000086470, and PubChem CID: 11371962 were all predicted to have antiviral activity against hepatitis either generally or specifically B or C (**Supplementary Tables S2A, D, E, G**). ZINC000000899909, ZINC000001632866, and ZINC000015151344 were predicted to inhibit hepatitis C virus (HCV) internal ribosome entry site important for translation initiation in HCV (Dibrov et al., 2014).

Other antiviral activity predicted included compounds ZINC000000899909 and ZINC000000086470 against rhinoviruses with Pa values of 0.383 and 0.568 and Pi values of 0.111 and 0.009, respectively (**Supplementary Tables S2A, E**). ZINC000000086470 and ZINC000013378519 had predicted antiviral activity against herpes and influenza (**Supplementary Tables S2D, E**). PubChem CID: 11371962 had predicted antiviral activity against human coronavirus with Pa of 0.247 and Pi of 0.080.

There were several other predicted activities that could lead to viral inhibition. All top seven compounds were predicted as RelA expression inhibitors, JAK2 expression inhibitors, and Pin1 inhibitors (**Supplementary Tables S2A-G**). The nuclear factor κB (NF- κB) pathway is important to viruses because this pathway can be activated by detection of viral particles leading to an immune response (Takada et al., 2002). RelA inhibitors block RelA, in NF- κB and Sp1 sufficiently to inhibit HIV-1 replication and decrease HIV-1 transcription (Takada et al., 2002). The highest predicted activity for RelA expression inhibition from the seven compounds was ZINC000000086470 with a Pa of 0.647 and Pi of 0.003, and second was ZINC000000899909 with a Pa of 0.623 and Pi of 0.003.

JAK2 is a substrate for Abl family tyrosine kinases and Abl family tyrosine kinases are a known target for anti-MPXV drugs (Rabaan et al., 2023). MPXV and vaccinia virus (VACV) use a conserved mechanism described previously to move from cell to cell. For both viruses, enveloped virions are important as it has been previously shown that the formation of actin tails necessary for motility require Abl and Src family kinases, though only Abl kinases are needed for release of the enveloped virions (Reeves et al., 2005; Reeves et al., 2011). There has been success with imatinib mesylate in blocking this pathway in mice models successfully inhibiting viral exit of VACV (Reeves et al., 2005). Abl family tyrosine kinase inhibitors have also been reported to inhibit viral replication by interrupting viral DNA synthesis (Rabaan et al., 2023; Reeves et al., 2005). Since JAK2 is known to be activated by poxviruses and it is involved in this pathway important for viral replication and motility, inhibition of JAK2 expression is a good target to inhibit poxviruses (Rabaan et al., 2023; Reeves et al., 2011; Ahmed et al., 2009). JAK2 expression inhibitors are also useful in controlling inflammation caused by response to viral infection. MPXV infection experiments in cynomolgus macaques have reported fatality associated with high numbers of cytokines termed a ‘cytokine storm’ (Lum et al., 2022; Goff et al., 2011). This is not uncommon as similar aberrant immune responses occur in SARS-CoV-2 in human patients and Influenza A in mice studies (Gajjela and Zhou, 2022; Wang et al., 2020). SARS-CoV-2 in human patients when treated with JAK2 inhibitors resulted in preventing severe respiratory side effects resulting from viral infection with minimal impact on the hosts immune system (Gajjela and Zhou, 2022). Influenza A manipulation of JAK2 is vital for viral replication. In a study using JAK2 inhibitor, gingerenone, they were able to limit severe respiratory effects and prolong survival of mice (Wang et al., 2020). There is evidence that poxviruses can activate tyrosine kinases like JAK2 and that inhibition of JAK2 can alter cytokine signals protecting mice subjects from lethal VACV infection (Ahmed et al., 2009). The compound with the highest predicted JAK2 expression inhibition was ZINC000013378519 with a Pa of 0.902 and a Pi of 0.003. Also, with high predicted activity were compounds ZINC000001632866, ZINC000015151344, and ZINC000095486204 with Pa values of 0.860, 0.796, and 0.620 and Pi values of 0.004, 0.008, and 0.029, respectively.

Pin1 is a peptidylprolyl isomerase involved in activating several oncogenes and turning off tumor suppressors which make it a target for viruses including SARS-CoV-2, HIV, and hepatitis B (Kanna et al., 2022; Zhou et al., 2021; Hou et al., 2015). In SARS-CoV-2 and other viruses Pin1 aids in viral growth (Kanna et al., 2022). Pin1 has been shown to promote HIV uncoating, reverse transcription, and viral integration to the host genome, and upon Pin1 inhibition these steps are also inhibited (Hou et al., 2015). Pin1 in hepatitis B viral infection associates with hepatitis B X protein (HBx), a critical protein for viral transcription and replication (Zhou et al., 2021). ZINC000001632866 and ZINC000015151344 had the two highest predicted activities as Pin1 expression inhibitors with Pa values of 0.661 and 0.636 and Pi values of 0.011 and 0.013, respectively.

Compounds ZINC000000899909, ZINC000015151344, and ZINC000095486204 were predicted as APOA1 expression enhancers with Pa values of 0.443, 0.420, and 0.362 and Pi values of 0.047, 0.059, and 0.104, respectively. APOA1 is the gene encoding apolipoprotein A-I, a major component of high-density lipoprotein (HDL) (Singh et al., 1999; Srinivas et al., 1990). HDL has broad antiviral activity inhibiting viral entry into cells (Singh et al., 1999). Apolipoprotein A-I has been shown to limit cell fusion in HIV infected cells, in recombinant vaccinia virus infected CD4+ HeLa cells expressing HIV envelope protein, and herpes simplex virus all of which during viral infection decrease HDL levels (Srinivas et al., 1990; Owens et al., 1990). In herpes simplex virus, Apolipoprotein A-I was able to inhibit cell fusion at 1 µM concentrations (Srinivas et al., 1990).

Other targets for suggested anti-MPXV drugs to block viral replication include DNA or RNA polymerase and topoisomerase inhibitors (Rabaan et al., 2023). All compounds were predicted to have DNA or RNA polymerase inhibition and compounds ZINC000000086470, ZINC000095486204, and PC11371962 were predicted to inhibit topoisomerase I while compound ZINC000013378519 was predicted to inhibit both topoisomerase I and II (**Supplementary Tables S2A-G**). The predicted antiviral activities of the seven potential lead compounds had Pa > Pi and are worthy of further experimental validation *in vitro* (Jamkhande and Barde, 2014).

### 2.6 Compound sources and structural similarities to compounds with known biological activities

PubChem CID: 11371962 was structurally similar to tecovirimat with a score of 0.962. Tecovirimat has shown inhibitory activity against MPXV *in vitro* with an IC_50_ of 12.7 nM and in mice models with an EC_50_ of 0.008 µM against Zaire Central African clade MPXV isolates and an EC_50_ of 0.006 µM against MPXV isolates from the 2022 Canadian/West African clade (Frenois-Veyrat et al., 2022; Warner et al., 2022). Tecovirimat has limited cases of use in human treatment of MPXV but has shown efficacy in the treatment of MPXV (Mbrenga et al., 2022; Desai et al., 2022). Tecovirimat targets viral p37 and F13L phospholipase needed for enveloping the virus, in this study tecovirimat was used as a reference control, though it does not target A42R specifically (Sherwat et al., 2022; Desai et al., 2022; Mucker et al., 2013). PubChem CID: 11371962 had a higher predicted binding affinity for A42R than tecovirimat (**Table 2**).

Compound ZINC000000899909 or sojagol, is a natural compound that is extracted from *Glycine max* or soybeans. Soybean metabolites include different flavonoids, isoflavonoids, and coumarins that have been suggested to play different roles in antimicrobial activities in plants (Silva et al., 2021). ZINC000000899909 was predicted to have structural similarity to (+)-rutamarin alcohol with a score of 0.716. (+)-Rutamarin alcohol’s direct parent is psoralens and is classed as a coumarin (Ulubelen and Öztürk, 2006). (+)-Rutamarin alcohol is reported to target topoisomerase II, that is critical for viral replication (Xu et al., 2014). ZINC000000899909 was reported in PASS to have inhibitory activity of both topoisomerase I and II with Pa’s of 0.272 and 0.147 and Pi’s of 0.018 and 0.035, respectively. (+)-Rutamarin alcohol has effectively inhibited herpesvirus replication *in vitro* with an IC_50_ of 1.12 µM and herpes virion production with an EC_50_ of 1.62 µM (Xu et al., 2014). It also inhibited Epstein Barr virus DNA replication at IC_50_ 2.38 µM and virion production with an EC_50_ of 2.94 µM (Hassan et al., 2022). Other coumarins, novobiocin and coumermycin inhibit viral topoisomerase 1B with Ki values of 10-25 µM and 350 µM, respectively (Sekiguchi et al., 1996). Vaccinia topoisomerase 1B has enough differences from human topoisomerase 1B to be selective to the viral version and is a suggested target in poxviruses (Sekiguchi et al., 1996; Sliva and Schnierle, 2007).

Compound ZINC000015151344 or 4,18-Dihydroxy-2-oxatricyclo[13.3.1.13,7]icosa-1(18),3,5,7(20),15(19),16-hexaen-10-one, is a natural compound that can be obtained from the tree *Engelhardia roxburghiana*. Other compounds extracted from the leaves of *Engelhardia roxburghiana* include flavonoids with effects as anti-inflammatories, anti-proliferatives, and antioxidants (Xin et al., 2012). ZINC000015151344 was predicted to have structural similarity to zingerone and 5-pentyl-2-phenoxyphenol with scores of 0.813 and 0.802 respectively. Zingerone is a compound from ginger with high antioxidant activity along with other important properties to ease complications associated with viral infection including anti-inflammatory, antimicrobial, anticancer, and antidiarrhoeic effects (Ahmad et al., 2015). Oxidative stress is associated with several viruses including vaccinia virus that causes redox imbalances in its hosts to promote viral replication (Aydemir and Ulusu, 2022). There is support of antioxidant compounds reducing lung inflammation after influenza A and B infection. Terameprocol is an antioxidant with both antiviral and anti-inflammatory effects that *in vitro* inhibited viral yield in both cowpox and vaccinia virus (Aydemir and Ulusu, 2022). 5-pentyl-2-phenoxyphenol is an antibacterial compound (Shawon et al., 2021). Chemically ZINC000015151344 is classified as a diarylether under the broader diarylheptanoids and 5-pentyl-2-phenoxyphenol’s direct parent is diphenyl ethers. Diphenyl ethers are of interest as new antiviral scaffolds (Kini et al., 2019). Diphenyl ether-based compounds have shown broad antiviral activity including efficacy against vaccinia virus *in vitro* with an EC_50_ of 9 µM (Ibrahim et al., 2016).

Compound ZINC000000086470 or Obovatin 5-Methyl Ether, was predicted to have structural similarity to sakuranetin, naringenin, (2S)-7-hydroxyflavanone, 5-deoxyflavanone, hesperetin, and 4’-hydroxyflavanone with scores 0.747, 0.742, 0.74, 0.736, 0.73, and 0.709, respectively. ZINC000000086470 can be found naturally in several species including *Tephrosia bracteolata*, *Lonchocarpus costaricensis*, and *Pongamia pinnata*. Extracts from *Tephrosia bracteolata* have shown antidiabetic, antioxidant, and antimicrobial properties (Egharevba et al., 2019). *Pongamia pinnata* is a species of tree with a wide variety of medicinal applications including uses as an antiseptic, and for the treatment of ulcers, malaria, bronchitis, and many more (Al Muqarrabun et al., 2013). More than seven different flavonoids have been extracted from *Lonchocarpus costaricensis*. Flavonoids are compounds in the flavanone class that are suggested to have broad antiviral activities (Jannat et al., 2021). Flavanoid compounds can inhibit a multitude of viral targets affecting viral binding, entry, and replication (Jannat et al., 2021). Sakuranetin has shown inhibitory activity against Influenza B replication with an IC_50_ of 7.21 µg/mL (Kwon et al., 2018). Hesperetin has antiviral activity against Sindbis neurovirulent strain with IC_50_ of 20.5 µg/mL (Paredes et al., 2003).

Compound ZINC000013378519 or 1-[(7-Hydroxy-4-methoxy-9,10-dihydrophenanthren-2-yl)oxy]-4-methoxy-9,10-dihydrophenanthrene-2,7-diol, is a natural product that can be obtained from members of the medicinal orchids, *Pholidota chinensis*, or *Bletilla striata*. Both orchid sources have a variety of known medicinal applications. *P. chinensis* has been used for the treatment of chronic bronchitis, fevers, stomachaches, ulcers, and has shown some anti-inflammatory effects in response to bacterial infections (Martha and Gutierrez, 2010). *B. striata* has shown activity as an antimicrobial agent, antioxidant, an anticancer agent, an anti-inflammatory, and has been used in wound healing and as a hemostatic agent (Martha and Gutierrez, 2010). The predicted biological activities of the shortlisted compounds corroborate their potential antiviral activity against MPXV. These 7 compounds are attractive antiviral candidates for *in vitro* experimentation.

### 2.7 Molecular dynamics simulations

To describe the structural conformation changes and atomic motions, 100 ns MD simulations were carried out for the unbound A42R, and A42R complexes with ligands ZINC000000899909, ZINC000001632866, ZINC000015151344, ZINC000013378519, ZINC000000086470, ZINC000095486204, and PC11371962. The top seven compounds used for MD had the highest binding affinities for A42R while binding to pocket 1, passed ADME, and had reasonably good predicted antiviral biological activity. Of the top compounds ZINC000000899909, ZINC000015151344, ZINC000013378519, ZINC000000086470, and PC11371962 also passed toxicity screening, where compounds ZINC000001632866 and ZINC000095486204 failed. ZINC000001632866 was specifically predicted to have antiviral activity against poxviruses. Since both compounds had potential for A42R specific inhibition and had high binding affinities, their ligand-protein interactions were of interest as they could be used as scaffolds or for optimization for future drug design, they were included in the MD simulations.

#### 2.7.1 RMSD of A42R and A42R-ligand complexes

**Figure 2:**
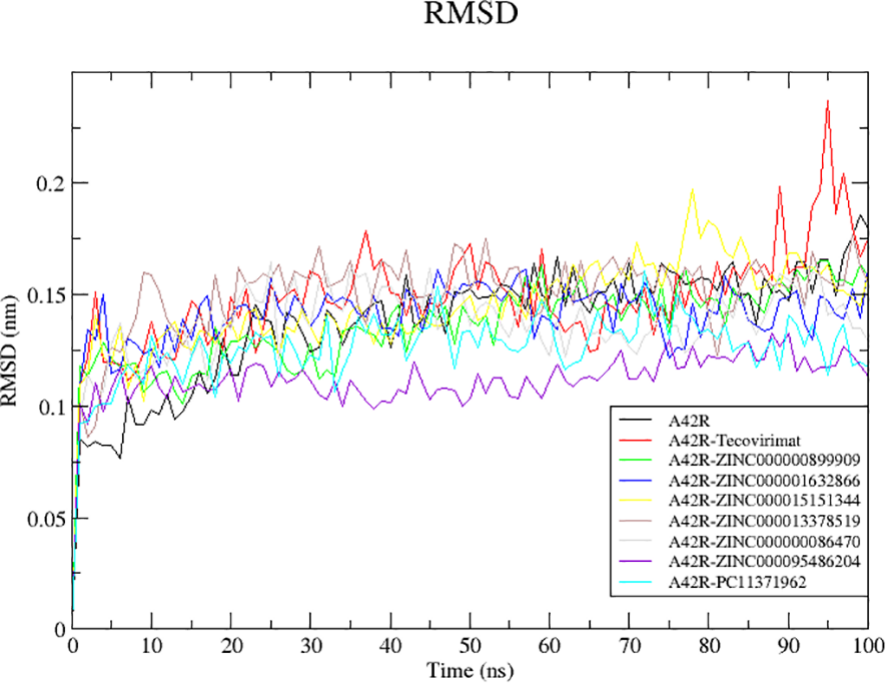
RMSD plot of the unbound A42R protein and A42R-ligand complexes. The unbound A42R protein, A42R complexes with tecovirimat, ZINC000000899909, ZINC000001632866, ZINC000015151344, ZINC000013378519, ZINC000000086470, ZINC000095486204, and PC11371962 are colored black, red, green, blue, yellow, brown, grey, violet, and cyan, respectively.

Since RMSD fluctuations are related to changes in the protein’s backbone, it is a good measurement of protein stability through the simulation (Adelusi et al., 2022). Low RMSD values correspond to more stability and high deviations represent less stability (Mangat et al., 2022; Bell and Zhang, 2019). All structures reached equilibrium by 20 ns (**Figure 2**). Unbound A42R remained mostly stable with few fluctuations from 20 ns until the end of the simulation with an average RMSD of 0.1378 nm (**Figure 2**). A42R complexes with compounds ZINC000095486204, PC11371962, ZINC000000899909, and ZINC000000086470 were more stable than the unbound protein during the simulation, with average RMSD values of 0.1116, 0.1258, 0.1359, and 0.1368 nm, respectively (**Figure 2**). The other A42R complexes had RMSD averages less than 2 Å corroborating the stability of the complexes (Ramírez and Caballero, 2018). All compounds except ZINC000013378519 had RMSD averages lower than tecovirimat’s RMSD average of 0.1482 (**Figure 2**).

A42R-ZINC000095486204 complex reached equilibrium quickly at around 5 ns and remained stable throughout the simulation with little fluctuation reflected in its low RMSD average of 0.1116. A42R-PC11371962 was mostly stable throughout the simulation. It reached equilibrium around 10 ns and had only moderate fluctuations until the end of the simulation (**Figure 2**). The A42R-ZINC000000899909 complex reached equilibrium around 20 ns and rose gradually from 30 to 50 ns then maintained stability for the rest of simulation (**Figure 2**). A42R-ZINC000000086470 reached equilibrium at around 15 ns and then rose at 20 ns to about 0.150 nm until 70 ns where it fell back to around 0.132 nm for the rest of the simulation (**Figure 2**).

The least stable complexes with A42R were A42R-tecovirimat and A42R-ZINC000013378519. The A42R-tecovirimat complex had a few spikes at 35, 85, and 95 ns jumping to 0.1810, 0.1985, and 0.2370 nm, respectively (**Figure 2**). The A42R-ZINC000013378519 complex had a small peak at 10 ns before equilibrating around 15 ns. It then maintained until about 80 ns where it dropped briefly and then rose back up for the rest of the simulation (**Figure 2**). The A42R-ZINC000015151344 complex equilibrated around 15 ns and maintained stability until around 55 ns where it began to rise and then spiked around 80 ns (**Figure 2**). The A42R-ZINC000001632866 complex equilibrated around 15 ns and maintained stability until 60 ns where it began to decline and then rose again around 80 ns until the end of simulation (**Figure 2**).

#### 2.7.2 Radius of gyration of A42R and A42R-ligand complexes

**Figure 3:**
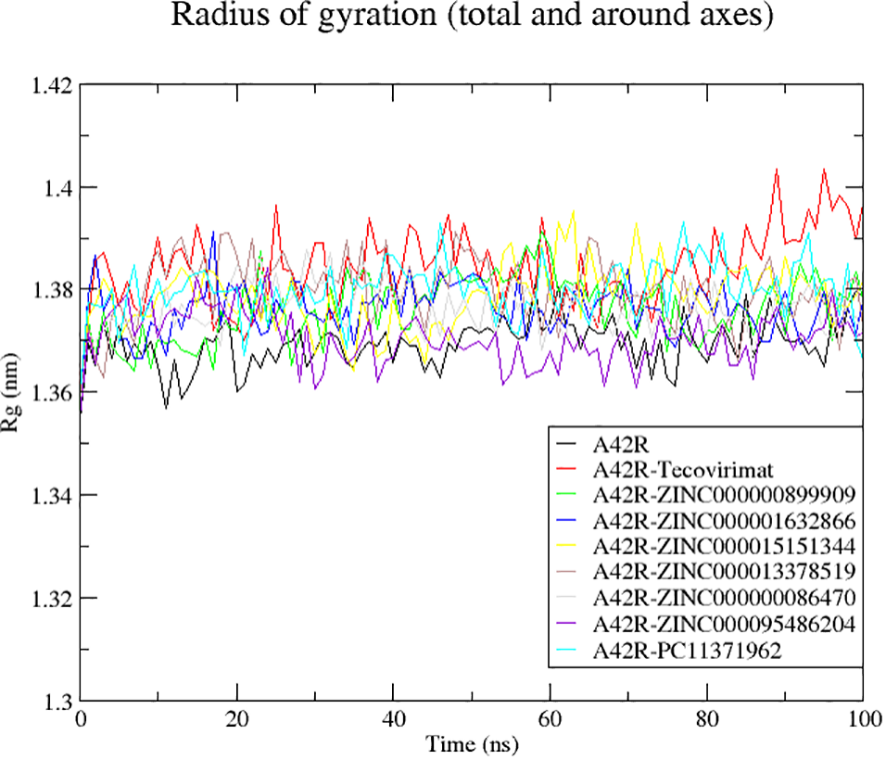
Radius of gyration plot of the unbound A42R protein and A42R-ligand complexes. The unbound A42R protein, A42R in complex with tecovirimat, ZINC000000899909, ZINC000001632866, ZINC000015151344, ZINC000013378519, ZINC000000086470, ZINC000095486204, and PC11371962 are colored black, red, green, blue, yellow, brown, grey, violet, and cyan, respectively.

Radius of gyration (Rg) is useful in evaluating stability, folding, and compactness of a protein (Lobanov et al., 2008; Ivankov et al., 2009). Rg is also known as the RMSD of atoms from the centroid of a protein (De Vita et al., 2021). Rg plots of the A42R unbound and A42R-ligand complexes correspond to good stability and compactness of A42R. Of all the Rg values plotted, they stayed between 1.3567 and 1.4044 nm and are considered stable folding (Lobanov et al., 2008). Unbound A42R had an average Rg of 1.3694 nm and all A42R-ligand complexes had similar averages ranging from 1.3705 and 1.3841 nm. The A42R-ZINC000095486204 complex had the lowest Rg average of the complexes at 1.3705 nm. It dropped around 20 ns and then remained with few fluctuations for the remainder of the 100 ns (**Figure 3**). A42R-ZINC000000899909, A42R-ZINC000001632866, and A42R-ZINC000000086470 complexes had Rg averages of 1.3762, 1.3760, and 1.3761 nm, respectively. The A42R-ZINC000000899909 complex rose around 20 ns and then dropped back around 60 ns (**Figure 3**). A42R-ZINC000001632866 spiked at 18 ns but then had few fluctuations for the remainder of the simulation (**Figure 3**). A42R-ZINC000000086470 rose slightly between 20 ns and 40 ns and then maintained small fluctuations for the remainder of the simulation (**Figure 3**). The Rg averages of the remaining compounds were 1.3841, 1.3799, 1.3784, and 1.3798 nm for A42R-tecovirimat, A42R-PC11371962, A42R-ZINC000015151344, and A42R-ZINC000013378519 complexes, respectively. The A42R-PC11371962 complex was mostly stable but rose at 50 ns and then came back down at 70 ns staying stable for the remainder of the simulation (**Figure 3**). A42R-ZINC000015151344 complex had slightly larger fluctuations but was otherwise stable for the 100 ns (**Figure 3**). The A42R-ZINC000013378519 complex rose quickly from 5 to 10 ns, dropping around 50 ns before fluctuating around 1.3745 nm for the remainder (**Figure 3**). A42R-tecovirimat had the largest fluctuation, though it was still not very large. A42R-tecovirimat had some larger fluctuations compared to the other compounds between 10 ns and 50 ns and then had a relatively large rise around 80 ns until the remainder of the simulation (**Figure 3**). Other studies that have carried out MD simulations on A42R-ligand complex have resulted in comparable or higher RMSD values (Bajrai et al., 2022; Burkhanova et al., 2022).

#### 2.7.3 RMSF of A42R-ligand complexes

RMSF of the unbound protein and its complexes were assessed to better understand residue interactions between the protein binding pocket and ligand (De Vita et al., 2021). RMSF also describes residues involved in mobility of the RMSD plots (De Vita et al., 2021). Low RMSF values indicate residues with strong interactions as they stay stable compared to high RMSF values that indicate weaker interactions characterized by higher mobility (De Vita et al., 2021).

**Figure 4:**
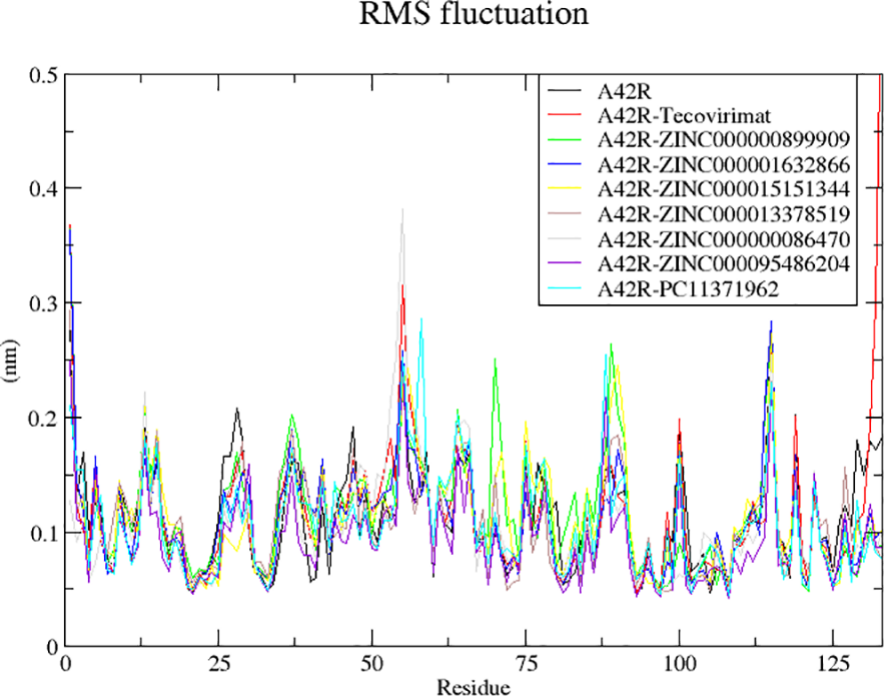
RMSF plot of the unbound A42R protein and A42R-ligand complexes. The unbound A42R protein, A42R in complex with tecovirimat, ZINC000000899909, ZINC000001632866, ZINC000015151344, ZINC000013378519, ZINC000000086470, ZINC000095486204, and PC11371962 are colored black, red, green, blue, yellow, brown, grey, violet, and cyan, respectively.

Fluctuations in RMSF were similar among the complexes (**Figure 4**). There were large fluctuations between residues 53-58, 87-93, and 96-112 (**Figure 4**). The highest fluctuations resulted from A42R-ZINC000000899909, A42R-ZINC000000086470, A42R- PC11371962, and A42R-tecovirimat. A42R-tecovirimat induced high fluctuations at residue His55 at 0.3139 nm, residue His100 at 0.2001 nm, residue Arg119 at 0.2010 nm, and the highest fluctuation at residue Gly132 at 0.5941 nm (**Figure 4**). A42R-ZINC000000899909 had high fluctuations at residue Tyr70 at 0.25 nm and residue Tyr88 at 0.2631 nm (**Figure 4**). A42R-ZINC000000086470 had a high fluctuation at residue His55 at 0.3804 nm (**Figure 4**). The A42R-PC11371962 complex had a high fluctuation at residue Leu58 at 0.2841 nm and residue Ala89 at 0.2640 nm (**Figure 4**). More minor fluctuations occurred around 26-30 and 38-53 (**Figure 4**). Residues experiencing minimal fluctuations were 3-20, 74-80, and 118-122, suggesting residues in these areas may interact strongly with the protein. Residues Trp4 and Ile7 had low RMSF values of 0.05655 nm and 0.6530 nm respectively. Residue Ala20 had the lowest RMSF value of 0.0452 nm. Residue Thr120 also had minimal fluctuation and a low RMSF of 0.04779 nm (**Figure 4**).

#### 2.7.4 Snapshot generation

To verify the position of the ligands during the simulation, snapshots were generated at 25 ns intervals. For each complex the ligand was bound to binding pocket 1 of A42R. Using structural alignment of the snapshots to the initial structure further confirmed the stability of the complexes during the MD simulation. RMSDs were then calculated for the alignment of each A42R complex using the align module in PyMOL.

The RMSD values for A42R-ZINC000095486204 snapshots at 25 ns, 50 ns, 75 ns, and 100 ns as aligned to the initial structure at 0 ns were 0.903, 0.839, 1.148, and 1.406 Å, respectively. A42R-PC11371962 snapshots generated RMSD values of 1.063, 1.015, 0.888, and 0.829 Å, respectively. The low RMSD values for A42R-ZINC000095486204 and A42R-PC11371962 are consistent with the RMSD plots (**Figure 2**). A42R-ZINC000000086470 snapshots generated RMSD values 1.25, 1.198, 1.923, and 1.247 Å, at 75 ns, respectively which are consistent with a minor fluctuation noted previously (**Figure 2**). A42R-ZINC000000899909 snapshots generated RMSD values of 0.817, 1.524, 1.112, and 1.411 Å, respectively. A42R-ZINC000001632866 snapshots generated RMSD values of 1.106, 1.629, 0.855, and 1.389 Å, respectively when the structures at 25, 50, 75, and 100 ns were aligned to the initial structure. A42R-ZINC000015151344 snapshots generated RMSD values of 1.028, 1.284, 1.305, and 1.168 Å where the 75 ns RMSD value reflects the spike seen (**Figure 2**). A42R-ZINC000013378519 snapshots generated RMSD values of 1.405, 1.129, 1.406, and 1.497 Å. A42R-tecovirimat snapshots generated RMSD values of 1.234, 1.267, 1.006, and 1.026 Å. The time of snapshots did not overlap with the major spike shown in the RMSD plot for A42R-tecovirimat (**Figure 2**). For all these A42R-ligand complex snapshots, they are relatively low RMSD values supporting the stability of these complexes as previously described in observations from the RMSD plots (**Figure 2**).

#### 2.7.5 Hydrogen bond analysis

Hydrogen bonds (H-bonds) between A42R and ligand during the 100 ns MD simulations were monitored using GROMACS “gmx hbond” (**Supplementary Figure S3**). H-bonds contribute to protein-ligand binding when the donor and acceptor have greater or lesser hydrogen bonding ability than the hydrogen or oxygens of water (Chen et al., 2016). H-bond pairings should be considered for further optimization of these compounds. Only A42R-ZINC000001632866 complex showed no H-bonds predicted from “gmx hbond”, this was supported by the visualization of LigPlot+, that also showed no H-bonds (**Supplementary Figures S2C, S3**). A42R- ZINC000000086470 began the simulation with 1 H-bond, which was lost rapidly, the H-bond was only recovered briefly from 45 ns to 70 ns before it was lost for the remainder of the simulation (**Supplementary Figure S3**). A42R-tecovirimat started and ended the simulation with 2 H-bonds, but for the majority of the simulation only produced 1 H-bond (**Supplementary Figure S3**). A42R-ZINC000015151344 formed the most hydrogen bonds during the MD simulations. A42R-ZINC000015151344 started with only 1, but eventually reached four H-bonds around 75 ns. A42R-ZINC000015151344 also maintained the highest amount throughout the simulation, for most of the MD simulation, it retained at least two H-bonds (**Supplementary Figure S3**). A42R- ZINC000000899909 managed to produce 3 H-bonds at around 20 ns, but for the majority of the simulation only maintained 1 H-bond (**Supplementary Figure S3**). A42R-ZINC000013378519 fluctuated between 1 and 2 H-bonds throughout the simulation. A42R-ZINC000095486204 for the majority retained 1 or 2 H-bonds, all H-bonds were lost around 70 ns (**Supplementary Figure S3**). A42R-PC11371962 started the simulation with 2 H-bonds and at least 1 H-bond for almost the entire simulation excluding from 10 ns to 19 ns and few drops in between was retained (**Supplementary Figure S3**).

### 2.8 MM/PBSA calculation of binding free energies and per residue energy contributions

Free binding energies and other energy contributors namely *van der Waals* (vdW), electrostatic, polar solvation and solvent accessible surface area (SASA) energies were also calculated using MM/PBSA (**Table 4**) (Kumari et al., 2014). The vdW energy of the A42R-ligand complexes ranged between –98.944 and –144.534 kJ/mol, where A42R-ZINC000000086470 had the least negative vdW energy and A42R-ZINC000013378519 displayed the most negative vdW energy (**Table 4**). The second most negative vdW energy was A42R-ZINC000001632866 with –125.513 kJ/mol followed by A42R-ZINC000000899909 with –121.779 kJ/mol. SASA energies ranged from –12.646 to –17.900 kJ/mol where A42R-tecovirimat had the least negative SASA energy and A42R-ZINC000013378519 had the most negative SASA energy (**Table 4**). The SASA energy has a linear relationship to non-polar solvation energy and differs minimally between structurally similar ligands (Kumari et al., 2014; Kollman et al., 2000; Genheden and Ryde, 2015).

**Table 4:** Contributing energy terms for the protein-ligand complexes determined via MM/PBSA calculations.

Compound	Van der Waals	Electrostatic Energy	Polar Solvation Energy	SASA Energy	Binding Energy
Tecovirimat	-103.240 ± 1.282	-25.951 ± 1.204	73.090 ± 1.699	-12.646 ± 0.106	-68.694 ± 1.198
PC-11371962	-104.257 ± 1.653	-24.260 ± 0.947	66.086 ± 1.851	-13.094 ± 0.176	-75.443 ± 1.517
ZINC000000899909	-121.779 ± 1.060	-24.517 ± 0.878	62.516 ± 1.318	-13.387 ± 0.106	-97.140 ± 1.443
ZINC000001632866	-125.513 ± 1.372	-16.293 ± 0.877	61.733 ± 1.234	-14.076 ± 0.086	-94.219 ± 1.318
ZINC000015151344	-114.491 ± 1.193	-30.548 ± 1.408	85.817 ± 1.558	-14.043 ± 0.063	-73.252 ± 1.186
ZINC000013378519	-144.534 ± 1.377	-10.922 ± 1.353	85.748 ± 2.210	-17.900 ± 0.133	-87.652 ± 1.578
ZINC000000086470	-98.844 ± 1.690	-6.989 ± 0.954	43.996 ± 1.662	-12.718 ± 0.164	-74.667 ± 1.461
ZINC000095486204	-106.888 ± 1.531	-19.915 ± 2.504	67.250 ± 3.405	-14.681 ± 0.158	-74.196 ± 1.416

All energy values are in kJ/mol. The energy values are presented as “energy ± standard deviation”.

A42R-tecovirimat had the highest binding energy at –68.694 kJ/mol (**Table 4**). Tecovirimat is a known inhibitor of MPXV, inhibiting p37 and F13L phospholipase and has not been shown to bind A42R (Sherwat et al., 2022; Mbrenga et al., 2022; Desai et al., 2022; Mucker et al., 2013). The other seven ligands had lower binding energies than tecovirimat suggesting that they might have higher affinities for A42R (**Table 4**). The compound with the lowest binding energy was ZINC000000899909 at –97.140 kJ/mol and close behind was ZINC000001632866 at –94.219 kJ/mol and ZINC000013378519 at –87.652 kJ/mol (**Table 4**). PC-11371962, was structurally similar to tecovirimat, but had a higher affinity for A42R with a binding energy of –75.443 kJ/mol (**Table 4**). ZINC000000086470, ZINC000095486204, and ZINC000015151344 all had comparable binding energies with –74.667, –74.196, and –73.252 kJ/mol, respectively (**Table 4**). These compounds have relatively good binding energies for A42R and provide rationale for *in vitro* validation of their inhibitory activity against MPXV, as well as other poxviruses. Compounds ZINC000001632866 and ZINC000095486204 did not pass toxicity screening but can be used as scaffolds and for information on A42R binding to aid in drug development targeting A42R, as they had low binding energies.

#### 2.8.1 Per-residue energy decomposition

**Figure 5:**
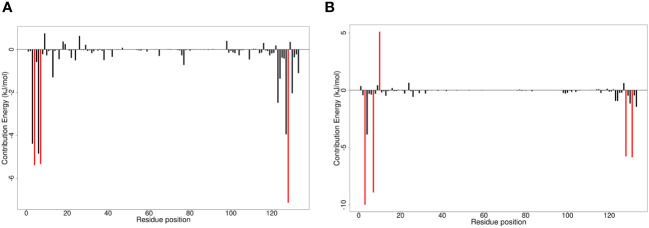
Molecular mechanics Poisson-Boltzmann surface areas (MM/PBSA) charts of per residue binding free energy contributions for **(A)** A42R-ZINC000000899909 and **(B)** A42R-ZINC000013378519 complexes. Critical residue fluctuations are colored red.

The g_mmpbsa tool was used to calculate the per residue energy contribution between A42R and the corresponding ligand (Kumari et al., 2014). Residues with contributing energies greater than 5 or less than –5 kJ/mol suggest residues important for protein-ligand interactions and should be considered for lead optimization (Kwofie et al., 2019). Per residue energy contribution charts were generated for each A42R-ligand complex (**Figure 5**; **Supplementary Figure S4**).

**Figure 6:**
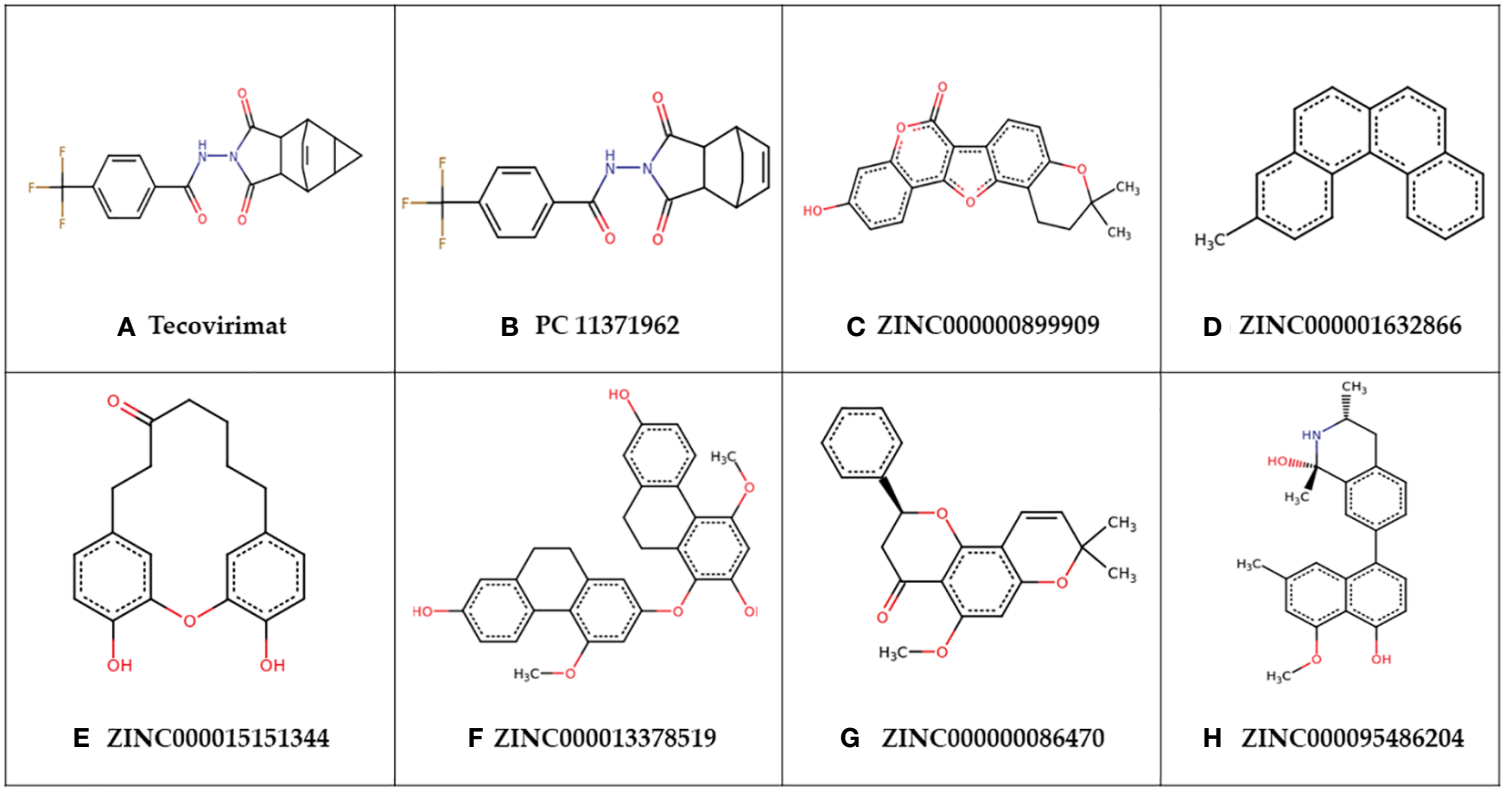
Chemical structures of **(A)** tecovirimat and **(B–H)** the top seven identified compounds.

Critical residues identified by per residue energy contributions included Trp4, Ile7, and Val128. Trp4 contributed over 5 kJ/mol for interactions between A42R and ligands ZINC000000899909, ZINC000000086470, and ZINC000095486204, with energy contributions of –5.2857, –7.5260, and –6.0570 kJ/mol, respectively (**Figures 5A**; **Supplementary Figure S4C, D**). Also, for the other complexes while not over the –5 kJ/mol threshold, Trp4 contributed between the range of –3.9842 to –4.7901 kJ/mol for all other complexes (**Figures 5B**; **Supplementary Figure S4A, B, E, F**). Ile7 was another critical residue greatly contributing to interactions in compounds ZINC000000899909, ZINC000001632866, ZINC000015151344, and ZINC000013378519 with energies of –5.3297, –5.7645, –7.7124, and –8.9099 kJ/mol, respectively (**Figure 5**; **Supplementary Figure S4A, B**). Ile7 was one of the highest contributors in PC-11371962 with an energy of –4.5854 kJ/mol, second only to Arg127 with an energy of –4.8324 kJ/mol (**Supplementary Figure S4E**). Val128 was a critical residue greatly contributing to interaction with A42R for compounds tecovirimat, ZINC000000899909, ZINC000001632866, ZINC000015151344, and ZINC000013378519 with energy contributions of –5.7186, –7.1385, –5.7766, –6.2818, and –5.7641 kJ/mol, respectively (**Figure 5**; **Supplementary Figure S4A, C, F**). Val128 was also contributing well for compounds ZINC000000086470, ZINC000095486204, and PC-11371962 with energies of –3.9444, –4.0847, and –4.1052 kJ/mol, respectively, though not past the –5 kJ/mol threshold (**Supplementary Figure S4C-E**). ZINC000013378519 had the greatest number of high energy contributions from residues Glu3, Trp4, Ile7, Asp10, Val128, and Thr131 (**Figure 5B**). Glu3 had a high contribution energy of –9.9873 kJ/mol, as did Thr131 of –5.8425 kJ/mol (**Figure 5B**). Asp10 contributed in the positive range with an energy contribution of 5.0947 kJ/mol (**Figure 5B**). The significant energy contributions of these residues make them interesting targets for future drug optimization. Chemical structures for the top seven compounds and tecovirimat are shown in **Figure 6**.

## 3 Materials and methods

**Figure 7:**
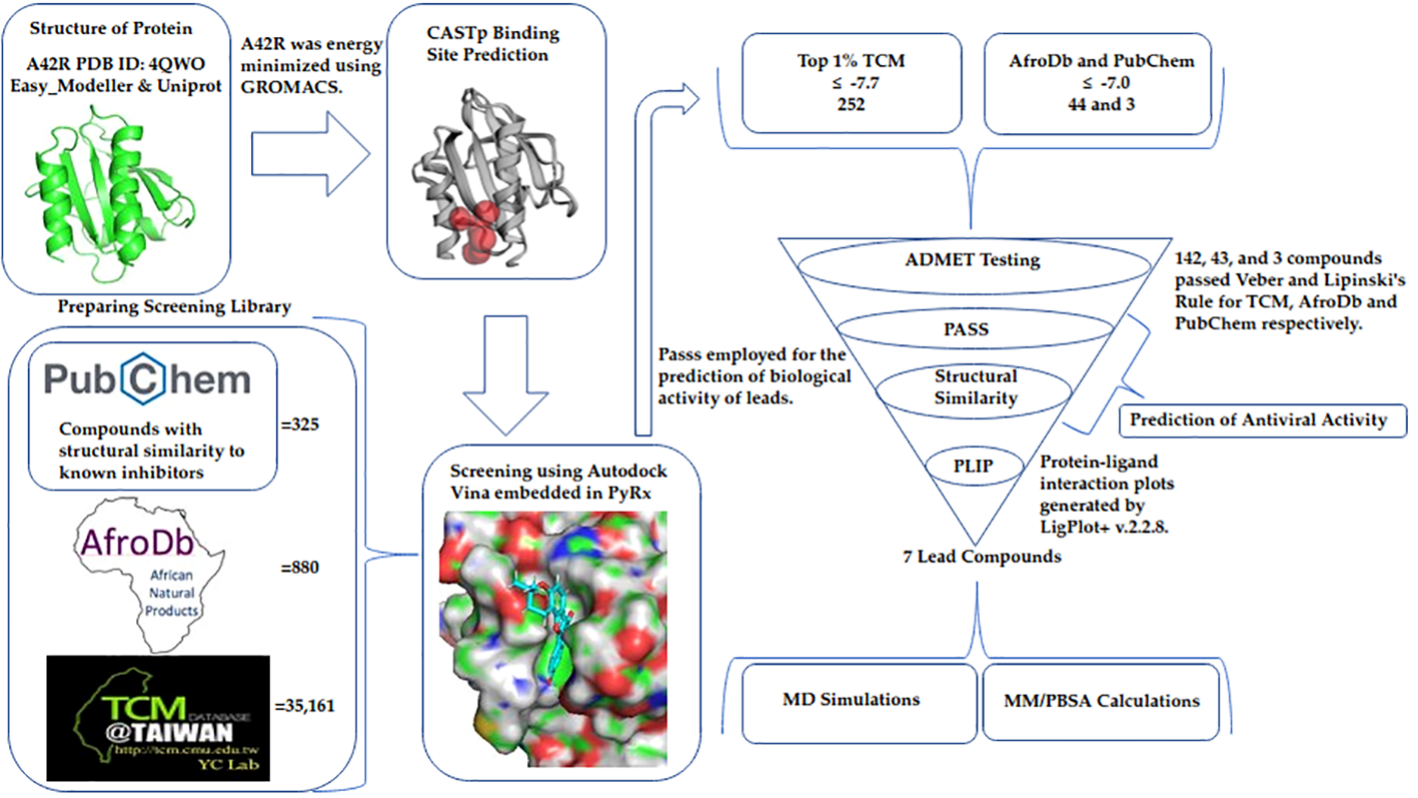
Method diagram detailing the process used in this study to identify potential A42R inhibitors.

A small molecule library of 36,366 compounds was screened for potential binding to MPXV protein A42R. The compounds with the highest affinity for A42R were then shortlisted using ADMET testing. Biological activity prediction and structural similarity searches were performed for the top compounds. MD simulations, protein-ligand interaction profiles, and MM/PBSA calculations were assessed for potential lead compounds to better understand the A42R-ligand interaction (**Figure 7**).

### 3.1 Drug target and binding site prediction

MPXV protein A42R (PDB ID: 4QWO) experimentally determined by X-ray diffraction with resolution of 1.52 Å, was retrieved from the RCSB PDB (Rose et al., 2017; Burley et al., 2021). The structure from RSCB PDB was in complex with ligands, ions, cofactors, and water molecules that were removed using PyMOL. The retrieved structure had missing residues; thus, chain A was remodeled using EasyModeller, a graphical user interface of Modeller. The complete A42R sequence was retrieved from UniProt with corresponding ID: Q8V4T7 (strain Zaire-96-I-16).

The remodeled structure had a discrete optimized protein energy (DOPE) score of –16024.13965, mol.pdf of 725.07245, and a genetic algorithm 341 (GA341) score of 1.0000. When aligned to the 4QWO structure, an RMSD of 0.092 was observed. GROningen MAchine for Chemical Simulations (GROMACS) v5.1.1 was used to energy minimize the protein structure using two different force fields namely, OPLS/AA and CHARMM36 force fields (Lu et al., 2021; Jo et al., 2008). This was done to compare and select the structure with the least energy after minimization. The A42R energy minimized protein in the GROMACS format (.gro) was then converted to pdb format after removing water and ions.

The binding sites of the A42R were predicted using CASTp 3.0. Usually, relatively large binding pockets correlate to an active site, though there are certainly exceptions (Liang et al., 1998). Predicted sites with relatively small cavity sizes were not considered further.

### 3.2 Collection and preparation of screening library

A screening library was generated to conduct structure-based virtual screening (SBVS) to identify potential binders of A42R. An integrated screening library comprised of 36,366 compounds from Traditional Chinese Medicine (TCM) database obtained from TCM@Taiwan, African Medical Plants (AfroDB), and PubChem (Chen, 2011; Ntie-Kang et al., 2013; Kim et al., 2023) was created. TCM and AfroDb are catalogues from the ZINC15 database (Sterling and Irwin, 2015). There were 35,161 compounds from TCM and 880 compounds from AfroDb. The 35,161 compounds from TCM were pre-filtered for compounds with molecular weights between 150 g/mol and 600 g/mol as done previously, leaving 25,196 compounds used from TCM (Kwofie et al., 2021). There were 325 compounds obtained from PubChem that were structurally similar to smallpox inhibitors tecovirimat, tembexa, and cidofovir. Tecovirimat was included in the library as a control because it has been FDA approved for the treatment of smallpox and has shown inhibition of MPXV (Adler et al., 2022; Frenois-Veyrat et al., 2022; Sherwat et al., 2022; Warner et al., 2022). Ligand structures originating from PubChem were downloaded in 3D spatial data file (.sdf) and merged with the compounds from AfroDb. All compound structures were imported into PyRx, energy minimized using the universal force field (UFF) and conjugate gradient algorithm in 200 steps, and then converted to pdbqt format (Rappe et al., 1992).

### 3.3 Molecular docking and protocol validation

AutoDock Vina (embedded in PyRx version 0.9.2), a docking program commonly used to perform protein-ligand docking, was used to screen the library for potential A42R binders and shortlist compounds for further assessment (Trott and Olson, 2010; Dallakyan and Olson, 2015). Docking used an exhaustiveness set to 8 with grid box dimensions of 37.964 × 20.791 × 28.223 Å^3^ and A42R centered at x = 30.406 Å, y = 22.08 Å, and z = 27.741 Å. The grid was made by setting the box to the following residues: Met1, Glu3, Trp4, Lys6, Ile7, Asp10, Ile22, Thr99, Ile104, His124, Ala125, Arg127, Val128, Thr131, and Asn133. For each ligand screened, AutoDock Vina generated up to 9 conformers. The poses were visualized using PyMOL to assert that the ligand was accurately bound in pocket 1 (the selected binding site). Due to the size disparity between the TCM database and the AfroDb and PubChem databases they were analyzed separately. The binding energy of -7.0 kcal/mol is a threshold specific to AutoDock Vina that separates putative binders and non-binders (Chang et al., 2007). The –7.0 cutoff has data to support that it filters around 95% of the non-inhibitors, but still passes about 98% of known inhibitors (Chang et al., 2007). The top 1% from the TCM ligands and all ligands below the –7.0 kcal/mol cutoff for AfroDB and PubChem were then shortlisted.

### 3.4 ADMET predictions of sub-library

To better characterize the shortlisted compounds’ pharmacokinetic profiles and drug-likeness they were ran through ADME testing via SwissADME (Daina et al., 2017). Shortlisted compounds were selected based on passing both Lipinski’s rule of five and Veber’s rule. The Lipinski’s rule of five requirement is met if the compound has one or less violations of the following rules: ≤ 5 hydrogen bond donors, ≤ 10 hydrogen bond acceptors, a molecular weight < 500 Da, and a lipophilicity or octanol-water partition coefficient (logP) ≤ 5 (Lipinski, 2016; Lipinski, 2004; Lipinski et al., 2001a; Lipinski et al., 2001b; Mullard, 2018). Veber’s rule requires ≤ 10 rotatable bonds and a topological polar surface area (TPSA) ≤ 140 Å^2^ (Veber et al., 2002).

To predict the toxic profiles of the compounds regarding potential mutagenicity, tumorigenicity, irritancy and reproductive effects, OSIRIS DataWarrior version 5.5.0 was used (Sander et al., 2015). DataWarrior predicts potential toxicities of compounds, classifying them as none, low, or high predicted risk for each property in question. Any compounds resulting in low or high toxicity regarding potential mutagenicity, tumorigenicity, or irritancy were removed from further consideration. Eliminating potential carcinogens was of importance because MPXV has been linked to increase tumor immunity and postulated to increase risk of tumor development (Liao et al., 2022). Also, commonly MPXV patients are coinfected with HIV (Hoffmann et al., 2022). HIV has been linked to an increased risk of cancer in what is termed AIDS-defining cancers (Grulich et al., 2007; Hernández-Ramírez et al., 2017). Potential toxicity regarding reproductive effects are reported but did not result in elimination of compounds for consideration.

### 3.5 Predictions of antiviral activity

Prediction of Activity Spectra of Substances (PASS) was used to predict biological activity of the shortlisted compounds. Of most interest was the compounds’ potential antiviral activity. PASS reads the SMILES format of the compound and then compares the structures of the molecules to its dataset comprised of active and inactive structural groups (Lagunin et al., 2000; Parasuraman, 2011; Filimonov et al., 2014). The read out for each compound is then a comparison of the probability of activity (Pa) to the probability of inactivity (Pi) where when Pa is greater than Pi the compound has the potential for that activity. To further corroborate potential activity, a similarity search for the shortlisted compounds was done using DrugBank to identify structural similarities with compounds that have experimentally validated antiviral activity (Wishart et al., 2008; Wishart et al., 2018).

### 3.6 Molecular dynamics simulations

GROMACS v5.1.1 was used for carrying out MD simulations (Abraham et al., 2017; Abraham et al., 2015). GROMACS software accuracy has been assessed in a comparison with experimental data supporting its usage for CADD (Childers and Daggett, 2018). Drug discovery relies on protein-ligand interactions where ligand binding has dynamic properties like flexibility and conformational changes that must be accounted for in drug design (Durrant and McCammon, 2011; De Vivo et al., 2016). MD simulations take into account the conformational changes and the movements associated with receptor-ligand binding interactions (Durrant and McCammon, 2011; De Vivo et al., 2016). These simulations are a computational method used to study the movement of atoms in a system using physics that modulates electric force changes in bonded and non-bonded atoms (Durrant and McCammon, 2011; Cheng and Ivanov, 2012). MD simulations still have limitations including sometimes necessary long simulation run times to accurately describe specific dynamic properties and insufficient mathematical models of forces influencing protein dynamics (Childers and Daggett, 2018). Even with limitations, MD simulations have supported research comparing simulation results with experimental data for their pertinent use in drug discovery (Adelusi et al., 2022; Childers and Daggett, 2018; Durrant and McCammon, 2011; De Vivo et al., 2016; Cheng and Ivanov, 2012).

To prepare ligands for MD, the ligand topologies for OPLS force field were created using LigParGen (Dodda et al., 2017). Solvation of the systems were made using a cubic box with the “TIP4P” water model and sodium or chlorine ions were added to neutralize charges (Lu et al., 2021; Nguyen et al., 2014). A42R-ligand systems prior to MD simulation were subjected to constant number, constant-volume and constant-temperature (NVT) and constant number, constant-pressure and constant-temperature (NPT). To evaluate the structural stability, folding and conformational fluctuations of A42R during MD simulations the RMSD, radius of gyration (Rg) and the root mean square fluctuation (RMSF) were calculated post simulation. At 25 ns intervals, snapshots were generated to ascertain the position of ligands with A42R.

### 3.7 Characterizing A42R-ligand interactions and MM/PBSA calculations

Interaction maps of the top seven compounds and tecovirimat with A42R were generated using LigPlot+. Hydrogen bonds during the MD simulations were monitored using GROMACS “gmx hbond”. Molecular interactions between the ligands and the A42R binding pocket are important to recognize for future studies as potential drug candidates.

MM/PBSA methods have been used successfully to reproduce experimental findings and are becoming more efficient and reliable methods for analyzing protein-ligand interactions (Genheden and Ryde, 2015; Sgobba et al., 2012). MM/PBSA performance evaluations have supported a higher yield of enrichment factors as compared to yields from docking scores alone and give reasonably accurate free energy calculations (Sgobba et al., 2012). MM/PBSA estimates the Gibbs free energy of binding, ΔG_(bind)_ of ligands to protein (Genheden and Ryde, 2015; Borkotoky et al., 2016). For drug discovery, the most negative ΔG_(bind)_ can be used to prioritize compounds for experimental trials (Sgobba et al., 2012). For this study the MM/PBSA approach was used to generate binding free energies and to compute the energy contributions per residue for each of the A42R-ligand complexes.

## 4 Conclusions

The recent dramatic spike in MPXV cases is reason for global concern. Transmission of MPXV between persons was previously referred to as limited however, smallpox infections and vaccinations have been shown to protect against MPXV and mathematical modelling in the context of decreasing herd immunity to orthopoxviruses indicates an increasing risk of disease spread between humans (Grant et al., 2020). Human transmission of MPXV leading to outbreaks in non-endemic areas has already been shown. To generate drugs for the defense against MPXV, this study shortlisted seven compounds from a library of 36,366 as potential anti-MPXV compounds targeting the A42R protein. These compounds had good predicted binding affinity to A42R from AutoDock Vina and from MM/PBSA calculations. All seven compounds have a higher predicted binding affinity to A42R than tecovirimat, a known MPXV inhibitor. MD simulations of the A42R-ligand complexes showed good stability and supported free binding energy results from MM/PBSA calculations. All seven compounds passed ADME screening and compounds ZINC000000899909, ZINC000015151344, ZINC000013378519, ZINC000000086470, and PC11371962 passed predicted toxicity screening. Predicted biological activity of the compounds supports their potential antiviral activity. Notably ZINC000001632866 and ZINC000015151344 were predicted as antivirals for poxviruses. Structural similarity with known antivirals (with anti-poxvirus activities) further supports the predicted biological activities of the shortlisted compounds. Compounds ZINC000001632866 and ZINC000095486204 failed toxicity screening and should not be considered candidates for further safety testing. It should also be considered that ZINC000001632866 and ZINC000013378519 were predicted not to cross the BBB and would require alternative administration. These three compounds may have functional groups of interest and support key contact residues within the A42R binding pocket that should be considered for future drug optimization. The identified compounds should be considered for *in vitro* validation of their efficacy against MPXV. These compounds may serve as scaffolds for MPXV drug design and future lead optimization.

## Data availability statement

The datasets presented in this study can be found in online repositories. The names of the repository/repositories and accession number(s) can be found in the article/**Supplementary Material**.

## Author contributions

CA: Formal analysis, Investigation, Methodology, Supervision, Validation, Writing – original draft, Writing – review & editing. EB: Formal analysis, Investigation, Methodology, Validation, Writing – original draft, Writing – review & editing. CW: Investigation, Methodology, Writing – original draft. TO: Investigation, Methodology, Writing – original draft. M-PO: Investigation, Methodology, Writing – review & editing. QD: Methodology, Project administration, Resources, Writing – review & editing. CG: Funding acquisition, Methodology, Project administration, Resources, Writing – review & editing. WM: Conceptualization, Funding acquisition, Project administration, Resources, Supervision, Writing – review & editing.

## References

[B1] Abraham M. J. Murtola T. Schulz R. Páll S. Smith J. C. Hess B.. (2015). GROMACS: High Performance Molecular Simulations through Multi-Level Parallelism from Laptops to Supercomputers (SoftwareX), 19–25. doi: 10.1016/J.SOFTX.2015.06.001

[B2] Abraham M. J. van der Spoel D. Lindahl E. Hess B. (2017). the GROMACS development team GROMACS User Manual Version 5.1.5.

[B3] Adelusi T. I. Oyedele A.-Q. K. Boyenle I. D. Ogunlana A. T. Adeyemi R. O. Ukachi C. D.. (2022). Molecular modeling in drug discovery. Inf. Med. Unlocked. 29, 100880. doi: 10.1016/j.imu.2022.100880

[B4] Adler H. Gould S. Hine P. Snell L. B. Wong W. Houlihan C. F.. (2022). Clinical features and management of human monkeypox: a retrospective observational study in the UK. Lancet Infect. Dis. 22, 1153–1162. doi: 10.1016/S1473-3099(22)00228-6 35623380 PMC9300470

[B5] Ahmad B. Rehman M. U. Amin I. Arif A. Rasool S. Bhat S. A.. (2015). Review on pharmacological properties of zingerone (4-(4-hydroxy-3-methoxyphenyl)-2-butanone). Sci. World J. 2015, 1–6. doi: 10.1155/2015/816364 PMC4461790 26106644

[B6] Ahmed C. M. Dabelic R. Waiboci L. W. Jager L. D. Heron L. L. Johnson H. M. (2009). SOCS-1 mimetics protect mice against lethal poxvirus infection: identification of a novel endogenous antiviral system. J. Virol. 83, 1402–1415. doi: 10.1128/JVI.01138-08 19019946 PMC2620917

[B7] Al Muqarrabun L. M. R. Ahmat N. Ruzaina S. A. S. Ismail N. H. Sahidin I.. (2013). Medicinal uses, phytochemistry and pharmacology of pongamia pinnata (L.) Pierre: a review. J. Ethnopharmacol 150, 395–420. doi: 10.1016/j.jep.2013.08.041 24016802

[B8] Antinori A. Mazzotta V. Vita S. Carletti F. Tacconi D. Lapini L. E.. (2022). Epidemiological, clinical and virological characteristics of four cases of monkeypox support transmission through sexual contact, Italy, May 2022. Eurosurveillance 27 (22), 2200421. doi: 10.2807/1560-7917.ES.2022.27.22.2200421 35656836 PMC9164671

[B9] Aydemir D. Ulusu N. N. (2022). The possible importance of the antioxidants and oxidative stress metabolism in the emerging monkeypox disease: An opinion paper. Front. Public Heal. 10. doi: 10.3389/fpubh.2022.1001666 PMC9633114 36339207

[B10] Bajrai L. H. Alharbi A. S. El-Day M. M. Bafaraj A. G. Dwivedi V. D. Azhar E. I. (2022). Identification of Antiviral Compounds against Monkeypox Virus Profilin-like Protein A42R from Plantago lanceolata. Molecules 27, 7718. doi: 10.3390/molecules27227718 36431817 PMC9697570

[B11] Bell E. W. Zhang Y. (2019). DockRMSD: an open-source tool for atom mapping and RMSD calculation of symmetric molecules through graph isomorphism. J. Cheminform 11, 40. doi: 10.1186/s13321-019-0362-7 31175455 PMC6556049

[B12] Billioux B. J. Mbaya O. T. Sejvar J. Nath A. (2022). Neurologic complications of smallpox and monkeypox: A review. JAMA Neurol. 79, 1180–1186. doi: 10.1001/jamaneurol.2022.3491 36125794

[B13] Borkotoky S. Meena C. K. Murali A. (2016). Interaction analysis of T7 RNA polymerase with heparin and its low molecular weight derivatives – an in silico approach. Bioinform. Biol. Insights 10, BBI.S40427. doi: 10.4137/BBI.S40427 PMC5004996 27594785

[B14] Broni E. Striegel A. Ashley C. Sakyi P. O. Peracha S. Velazquez M.. (2023). Molecular docking and dynamics simulation studies predict potential anti-ADAR2 inhibitors: implications for the treatment of cancer, neurological, immunological and infectious diseases. Int. J. Mol. Sci. 24, 6795. doi: 10.3390/IJMS24076795 37047766 PMC10095294

[B15] Bunge E. M. Hoet B. Chen L. Lienert F. Weidenthaler H. Baer L. R.. (2022). The changing epidemiology of human monkeypox—A potential threat? A systematic review. PloS Negl. Trop. Dis. 16 (2), e0010141. doi: 10.1371/journal.pntd.0010141 35148313 PMC8870502

[B16] Burkhanova T. M. Krysantieva A. I. Babashkina M. G. Konyaeva I. A. Monina L. N. Goncharenko A. N.. (2022). In silico analyses of betulin: DFT studies, corrosion inhibition properties, ADMET prediction, and molecular docking with a series of SARS-CoV-2 and monkeypox proteins. Struct. Chem. doi: 10.1007/s11224-022-02079-8 PMC9607775 36320318

[B17] Burley S. K. Bhikadiya C. Bi C. Bittrich S. Chen L. Crichlow G. V.. (2021). RCSB Protein Data Bank: powerful new tools for exploring 3D structures of biological macromolecules for basic and applied research and education in fundamental biology, biomedicine, biotechnology, bioengineering and energy sciences. Nucleic Acids Res. 49, D437–D451. doi: 10.1093/NAR/GKAA1038 33211854 PMC7779003

[B18] Butler-Cole C. Wagner M. J. Da Silva M. Brown G. D. Burke R. D. Upton C. (2007). An ectromelia virus profilin homolog interacts with cellular tropomyosin and viral A-type inclusion protein. Virol. J. 4, 76. doi: 10.1186/1743-422X-4-76 17650322 PMC1964790

[B19] Chakraborty S. Chandran D. Mohapatra R. K. Alagawany M. El-Shall N. A. Sharma A. K.. (2022). Clinical management, antiviral drugs and immunotherapeutics for treating monkeypox. An update on current knowledge and futuristic prospects. Int. J. Surg. 105, 106847. doi: 10.1016/j.ijsu.2022.106847 35995352 PMC9533875

[B20] Chang M. W. Lindstrom W. Olson A. J. Belew R. K. (2007). Analysis of HIV wild-type and mutant structures via in silico docking against diverse ligand libraries. J. Chem. Inf Model. doi: 10.1021/ci700044s 17447753

[B21] Chen C. Y.-C. (2011). TCM Database@Taiwan: the world’s largest traditional Chinese medicine database for drug screening in silico. PloS One 6, e15939. doi: 10.1371/journal.pone.0015939 21253603 PMC3017089

[B22] Chen D. Oezguen N. Urvil P. Ferguson C. Dann S. M. Savidge T. C. (2016). Regulation of protein-ligand binding affinity by hydrogen bond pairing. Sci. Adv. 2 (3), e1501240. doi: 10.1126/sciadv.1501240 27051863 PMC4820369

[B23] Cheng X. Ivanov I. (2012). Molecular dynamics. Methods Mol Biol. 929, 243–85. doi: 10.1007/978-1-62703-050-2_11 23007433

[B24] Childers M. C. Daggett V. (2018). Validating molecular dynamics simulations against experimental observables in light of underlying conformational ensembles. J. Phys. Chem. B 122, 6673–6689. doi: 10.1021/acs.jpcb.8b02144 29864281 PMC6420231

[B25] Cohen J. (2022). Monkeypox outbreak questions intensify as cases soar. Science 376, 902–903. doi: 10.1126/science.add1583 35617408

[B26] da Costa R. L. da Lamas C. Simvoulidis L. F. N. Espanha C. A. Moreira L. P. M. Bonancim R. A. B.. (2022). Secondary infections in a cohort of patients with COVID-19 admitted to an intensive care unit: impact of gram-negative bacterial resistance. Rev. Inst Med. Trop. Sao Paulo 64, e6. doi: 10.1590/S1678-9946202264006 35137900 PMC8815857

[B27] Daina A. Michielin O. Zoete V. (2017). SwissADME: a free web tool to evaluate pharmacokinetics, drug-likeness and medicinal chemistry friendliness of small molecules. Sci. Rep. 7, 42717. doi: 10.1038/srep42717 28256516 PMC5335600

[B28] Dallakyan S. Olson A. J. (2015). Small-molecule library screening by docking with PyRx. Methods in Molecular Biology. 1263, 243–250. doi: 10.1007/978-1-4939-2269-7_19 25618350

[B29] Dassanayake M. K. Khoo T.-J. Chong C. H. Di Martino P. (2022). Molecular docking and in-silico analysis of natural biomolecules against dengue, ebola, zika, SARS-coV-2 variants of concern and monkeypox virus. Int. J. Mol. Sci. 23, 11131. doi: 10.3390/ijms231911131 36232431 PMC9569982

[B30] de la Calle-Prieto F. Estébanez Muñoz M. Ramírez G. Díaz-Menéndez M. Velasco M. Azkune Galparsoro H.. (2023). Treatment and prevention of monkeypox. Enfermedades Infecc y Microbiol. Clin. doi: 10.1016/j.eimce.2022.12.010 PMC9823286 36624034

[B31] Desai A. N. Thompson G. R. Neumeister S. M. Arutyunova A. M. Trigg K. Cohen S. H. (2022). Compassionate use of tecovirimat for the treatment of monkeypox infection. JAMA 328, 1348–1350. doi: 10.1001/jama.2022.15336 35994281 PMC9396467

[B32] De Vita S. Chini M. G. Bifulco G. Lauro G. (2021). Insights into the ligand binding to bromodomain-containing protein 9 (BRD9): A guide to the selection of potential binders by computational methods. Molecules 26, 7192. doi: 10.3390/molecules26237192 34885774 PMC8659208

[B33] De Vivo M. Masetti M. Bottegoni G. Cavalli A. (2016). Role of molecular dynamics and related methods in drug discovery. J. Med. Chem. 59, 4035–4061. doi: 10.1021/acs.jmedchem.5b01684 26807648

[B34] Dibrov S. M. Parsons J. Carnevali M. Zhou S. Rynearson K. D. Ding K.. (2014). Hepatitis C virus translation inhibitors targeting the internal ribosomal entry site. J. Med. Chem. 57, 1694–1707. doi: 10.1021/jm401312n 24138284 PMC3954896

[B35] Dodda L. S. Cabeza de Vaca I. Tirado-Rives J. Jorgensen W. L. (2017). LigParGen web server: an automatic OPLS-AA parameter generator for organic ligands. Nucleic Acids Res. 45, W331–W336. doi: 10.1093/nar/gkx312 28444340 PMC5793816

[B36] Duncan M. L. Horsington J. Eldi P. Al Rumaih Z. Karupiah G. Newsome T. P. (2018). Loss of actin-based motility impairs ectromelia virus release in *vitro* but is not critical to spread in *vivo* . Viruses 10, 111. doi: 10.3390/v10030111 29510577 PMC5869504

[B37] Durrant J. D. McCammon J. A. (2011). Molecular dynamics simulations and drug discovery. BMC Biol. 9, 71. doi: 10.1186/1741-7007-9-71 22035460 PMC3203851

[B38] Egharevba G. O. Dosumu O. O. Oguntoye S. O. Njinga N. S. Dahunsi S. O. v A. A.. (2019). Antidiabetic, antioxidant and antimicrobial activities of extracts of tephrosia bracteolata leaves. Heliyon 5, e02275. doi: 10.1016/j.heliyon.2019.e02275 31485511 PMC6716168

[B39] Filimonov D. A. Lagunin A. A. Gloriozova T. A. Rudik A. V. Druzhilovskii D. S. Pogodin P. V.. (2014). Prediction of the biological activity spectra of organic compounds using the pass online web resource. Chem. Heterocycl Compd 50, 444–457. doi: 10.1007/s10593-014-1496-1

[B40] Frenois-Veyrat G. Gallardo F. Gorgé O. Marcheteau E. Ferraris O. Baidaliuk A.. (2022). Tecovirimat is effective against human monkeypox virus in *vitro* at nanomolar concentrations. Nat. Microbiol. 7, 1951–1955. doi: 10.1038/s41564-022-01269-8 36344621 PMC12536428

[B41] Gajjela B. K. Zhou M.-M. (2022). Calming the cytokine storm of COVID-19 through inhibition of JAK2/STAT3 signaling. Drug Discovery Today 27, 390–400. doi: 10.1016/j.drudis.2021.10.016 34743903 PMC8553370

[B42] Genheden S. Ryde U. (2015). The MM/PBSA and MM/GBSA methods to estimate ligand-binding affinities. Expert Opin. Drug Discovery 10, 449–461. doi: 10.1517/17460441.2015.1032936 PMC4487606 25835573

[B43] Gernert M. Feja M. (2020). Bypassing the blood–brain barrier: direct intracranial drug delivery in epilepsies. Pharmaceutics 12, 1134. doi: 10.3390/pharmaceutics12121134 33255396 PMC7760299

[B44] Goff A. J. Chapman J. Foster C. Wlazlowski C. Shamblin J. Lin K.. (2011). A novel respiratory model of infection with monkeypox virus in cynomolgus macaques. J. Virol. 85, 4898–4909. doi: 10.1128/jvi.02525-10 21389129 PMC3126178

[B45] Grant R. Nguyen L. B. L. Breban R. (2020). Modelling human-to-human transmission of monkeypox. Bull. World Health Organ 98, 638–640. doi: 10.2471/BLT.19.242347 33012864 PMC7463189

[B46] Grulich A. E. van Leeuwen M. T. Falster M. O. Vajdic C. M. (2007). Incidence of cancers in people with HIV/AIDS compared with immunosuppressed transplant recipients: a meta-analysis. Lancet 370, 59–67. doi: 10.1016/S0140-6736(07)61050-2 17617273

[B47] Hallo-Carrasco A. Hunt C. L. Prusinski C. C. Eldrige J. S. McVeigh K. H. Hurdle M. F. B.. (2023). Pain associated with monkeypox virus: A rapid review. Cureus. doi: 10.7759/cureus.34697 PMC9995223 36909034

[B48] Hassan S. T. S. Šudomová M. Mazurakova A. Kubatka P. (2022). Insights into Antiviral Properties and Molecular Mechanisms of Non-Flavonoid Polyphenols against Human Herpesviruses. Int. J. Mol. Sci. 23, 13891. doi: 10.3390/ijms232213891 36430369 PMC9693824

[B49] Heaton N. S. Randall G. (2011). Multifaceted roles for lipids in viral infection. Trends Microbiol. 19, 368–375. doi: 10.1016/j.tim.2011.03.007 21530270 PMC3130080

[B50] Hernández-Ramírez R. U. Shiels M. S. Dubrow R. Engels E. A. (2017). Cancer risk in HIV-infected people in the USA from 1996 to 2012: a population-based, registry-linkage study. Lancet HIV 4, e495–e504. doi: 10.1016/S2352-3018(17)30125-X 28803888 PMC5669995

[B51] Hersh D. S. Wadajkar A. S. Roberts N. Perez J. G. Connolly N. P. Frenkel V.. (2016). Evolving drug delivery strategies to overcome the blood brain barrier. Curr. Pharm. Des. doi: 10.2174/1381612822666151221150733 PMC4900538 26685681

[B52] Hoffmann C. Jessen H. Wyen C. Grunwald S. Noe S. Teichmann J.. (2022). Clinical characteristics of monkeypox virus infections among men with and without HIV: A large outbreak cohort in Germany. HIV Med. doi: 10.1111/hiv.13378 36059149

[B53] Hou H. Wang J.-Z. Liu B.-G. Zhang T. (2015). Pin1 liberates the human immunodeficiency virus type-1 (HIV-1): Must we stop it? Gene 565, 9–14. doi: 10.1016/j.gene.2015.04.049 25913034

[B54] Huang Y. A. Howard-Jones A. R. Durrani S. Wang Z. Williams P. C. M. (2022). Monkeypox: A clinical update for paediatricians. J. Paediatr. Child Health. doi:3 10.1111/jpc.16171 PMC9545589 35979896

[B55] Ibrahim T. S. AL-Mahmoudy A. M. M. Elagawany M. Ibrahim M. A. Panda S. S. (2016). Synthesis and antiviral bioassay of new diphenyl ether-based compounds. Chem. Biol. Drug Des. 88, 511–518. doi: 10.1111/cbdd.12775 27096302

[B56] Ivankov D. N. Bogatyreva N. S. Lobanov M. Y. Galzitskaya O. V. (2009). Coupling between properties of the protein shape and the rate of protein folding. PloS One 4, e6476. doi: 10.1371/journal.pone.0006476 19649298 PMC2714458

[B57] Jamkhande P. G. Barde S. R. (2014). Evaluation of anthelmintic activity and in silico PASS assisted prediction of Cordia dichotoma (Forst.) root extract. Anc Sci. Life 34, 39–43. doi: 10.4103/0257-7941.150779 25737609 PMC4342648

[B58] Jannat K. Paul A. K. Bondhon T. A. Hasan A. Nawaz M. Jahan R.. (2021). Nanotechnology applications of flavonoids for viral diseases. Pharmaceutics 13, 1895. doi: 10.3390/pharmaceutics13111895 34834309 PMC8625292

[B59] Jeon C. Y. Neidell M. Jia H. Sinisi M. Larson E. (2012). On the role of length of stay in healthcare-associated bloodstream infection. Infect. Control Hosp Epidemiol. 33, 1213–1218. doi: 10.1086/668422 23143358 PMC3510977

[B60] Jo S. Kim T. Iyer V. G. Im W. (2008). CHARMM-GUI: A web-based graphical user interface for CHARMM. J. Comput. Chem. 29, 1859–1865. doi: 10.1002/jcc.20945 18351591

[B61] Kabuga A. I. El Zowalaty M. E. (2019). A review of the monkeypox virus and a recent outbreak of skin rash disease in Nigeria. J. Med. Virol. 91, 533–540. doi: 10.1002/jmv.25348 30357851

[B62] Kandra N. V. Varghese A. M. Uppala P. K. Uttaravelli U. Lavanya B. Shabana S. K. M.. (2023). Monkeypox outbreak in the post-eradication era of smallpox. Egypt J. Intern. Med. 35, 10. doi: 10.1186/s43162-023-00196-2 36777901 PMC9897877

[B63] Kanna M. Nakatsu Y. Yamamotoya T. Encinas J. Ito H. Okabe T.. (2022). Roles of peptidyl prolyl isomerase Pin1 in viral propagation. Front. Cell Dev. Biol. 10. doi: 10.3389/fcell.2022.1005325 PMC9642847 36393854

[B64] Kim S. Chen J. Cheng T. Gindulyte A. He J. He S.. (2023). PubChem 2023 update. Nucleic Acids Res. 51, D1373–D1380. doi: 10.1093/nar/gkac956 36305812 PMC9825602

[B65] Kini S. G. Rathi E. Kumar A. Bhat V. (2019). Potentials of diphenyl ether scaffold as a therapeutic agent: A review. Mini-Reviews Med. Chem. 19, 1392–1406. doi: 10.2174/1389557519666190312150132 30864517

[B66] Kollman P. A. Massova I. Reyes C. Kuhn B. Huo S. Chong L.. (2000). Calculating structures and free energies of complex molecules: combining molecular mechanics and continuum models. Acc Chem. Res. 33, 889–897. doi: 10.1021/ar000033j 11123888

[B67] Koyuncu O. O. Hogue I. B. Enquist L. W. (2013). Virus infections in the nervous system. Cell Host Microbe 13, 379–393. doi: 10.1016/j.chom.2013.03.010 23601101 PMC3647473

[B68] Kumari R. Kumar R. Lynn A. (2014). g_mmpbsa —A GROMACS tool for high-throughput MM-PBSA calculations. J. Chem. Inf Model. 54, 1951–1962. doi: 10.1021/ci500020m 24850022

[B69] Kwofie S. Broni E. Yunus F. Nsoh J. Adoboe D. Miller W.. (2021). Molecular docking simulation studies identifies potential natural product derived-antiwolbachial compounds as filaricides against onchocerciasis. Biomedicines 9, 1682. doi: 10.3390/biomedicines9111682 34829911 PMC8615632

[B70] Kwofie S. Dankwa B. Enninful K. Adobor C. Broni E. Ntiamoah A.. (2019). Molecular docking and dynamics simulation studies predict munc18b as a target of mycolactone: A plausible mechanism for granule exocytosis impairment in buruli ulcer pathogenesis. Toxins (Basel) 11, 181. doi: 10.3390/toxins11030181 30934618 PMC6468854

[B71] Kwon D.-H. Ji J.-H. Yim S.-H. Kim B.-S. Choi H.-J. (2018). Suppression of influenza B virus replication by sakuranetin and mode of its action. Phyther Res. 32, 2475–2479. doi: 10.1002/ptr.6186 30187587

[B72] Lagunin A. Stepanchikova A. Filimonov D. Poroikov V. (2000). PASS: prediction of activity spectra for biologically active substances. Bioinformatics 16, 747–748. doi: 10.1093/bioinformatics/16.8.747 11099264

[B73] Li K. Yuan Y. Jiang L. Liu Y. Liu Y. Zhang L. (2023). Animal host range of mpox virus. J. Med. Virol. 95 (2), e28513. doi: 10.1002/jmv.28513 36661039

[B74] Liang J. Woodward C. Edelsbrunner H. (1998). Anatomy of protein pockets and cavities: Measurement of binding site geometry and implications for ligand design. Protein Sci. 7, 1884–1897. doi: 10.1002/pro.5560070905 9761470 PMC2144175

[B75] Liao Y. Liu Z. Ye W. Huang Z. Wang J. (2022). Exploring the characteristics of monkeypox-related genes in pan-cancer. Cells 11, 3909. doi: 10.3390/cells11233909 36497164 PMC9740123

[B76] Lipinski C. A. (2004). Lead- and drug-like compounds: The rule-of-five revolution. Drug Discovery Today Technol. doi: 10.1016/j.ddtec.2004.11.007 24981612

[B77] Lipinski C. A. (2016). Rule of five in 2015 and beyond: Target and ligand structural limitations, ligand chemistry structure and drug discovery project decisions. Adv. Drug Delivery Rev. 101, 34–41. doi: 10.1016/j.addr.2016.04.029 27154268

[B78] Lipinski C. A. Lombardo F. Dominy B. W. Feeney P. J. (2001a). Experimental and computational approaches to estimate solubility and permeability in drug discovery and development settings. Adv. Drug Delivery Rev. 46, 3–26. doi: 10.1016/S0169-409X(96)00423-1 11259830

[B79] Lipinski C. A. Lombardo F. Dominy B. W. Feeney P. J. (2001b). Experimental and computational approaches to estimate solubility and permeability in drug discovery and development settings1PII of original article: S0169-409X(96)00423-1. Adv. Drug Delivery Rev. 46, 3–26. doi: 10.1016/S0169-409X(00)00129-0 11259830

[B80] Lobanov M. Y. Bogatyreva N. S. Galzitskaya O. V. (2008). Radius of gyration as an indicator of protein structure compactness. Mol. Biol. 42, 623–628. doi: 10.1134/S0026893308040195 18856071

[B81] Lu C. Wu C. Ghoreishi D. Chen W. Wang L. Damm W.. (2021). OPLS4: improving force field accuracy on challenging regimes of chemical space. J. Chem. Theory Comput. 17, 4291–4300. doi: 10.1021/acs.jctc.1c00302 34096718

[B82] Lum F. M. Torres-Ruesta A. Tay M. Z. Lin R. T. P. Lye D. C. Rénia L.. (2022). Monkeypox: disease epidemiology, host immunity and clinical interventions. Nat. Rev. Immunol. 22, 597–613. doi: 10.1038/s41577-022-00775-4 36064780 PMC9443635

[B83] Machesky L. M. Cole N. B. Moss B. Pollard T. D. (1994). Vaccinia virus expresses a novel profilin with a higher affinity for polyphosphoinositides than actin. Biochemistry 33, 10815–10824. doi: 10.1021/bi00201a032 8075084

[B84] Mangat H. K. Rani M. Pathak R. K. Yadav I. S. Utreja D. Chhuneja P. K.. (2022). Virtual screening, molecular dynamics and binding energy-MM-PBSA studies of natural compounds to identify potential EcR inhibitors against Bemisia tabaci Gennadius. PloS One 17, e0261545. doi: 10.1371/journal.pone.0261545 35061725 PMC8782374

[B85] Martha R. Gutiérrez P.. (2010). Orchids: a review of uses in traditional medicine, its phytochemistry and pharmacology. Med. Plants Res. 4, 592–638. doi: 10.5897/JMPR10.012

[B86] Mbrenga F. Nakouné E. Malaka C. Bourner J. Dunning J. Vernet G.. (2022). Tecovirimat for monkeypox in Central African Republic under expanded access. N Engl. J. Med. 387, 2294–2295. doi: 10.1056/NEJMc2210015 36449745 PMC10117058

[B87] Meiser A. Sancho C. Krijnse Locker J. (2003). Plasma membrane budding as an alternative release mechanism of the extracellular enveloped form of vaccinia virus from heLa cells. J. Virol. 77, 9931–9942. doi: 10.1128/JVI.77.18.9931-9942.2003 12941903 PMC224582

[B88] Meo S. A. Ali Jawaid S. (2022). Human Monkeypox: Fifty-Two Years based analysis and Updates. Pakistan J. Med. Sci. 38 (6), 1416–1419. doi: 10.12669/pjms.38.6.6775 PMC9378419 35991265

[B89] Miller M. J. Cash-Goldwasser S. Marx G. E. Schrodt C. A. Kimball A. Padgett K.. (2022). Severe monkeypox in hospitalized patients — United states, august 10–october 10, 2022. MMWR Morb Mortal Wkly Rep. 71, 1412–1417. doi: 10.15585/mmwr.mm7144e1 36327164 PMC9639440

[B90] Minasov G. Inniss N. L. Shuvalova L. Anderson W. F. Satchell K. J. F. (2022). Structure of the Monkeypox virus profilin-like protein A42R reveals potential functional differences from cellular profilins. Acta Crystallogr. Sect F Struct. Biol. Commun. 78, 371–377. doi: 10.1107/S2053230X22009128 36189721 PMC9527652

[B91] Mucker E. M. Goff A. J. Shamblin J. D. Grosenbach D. W. Damon I. K. Mehal J. M.. (2013). Efficacy of tecovirimat (ST-246) in nonhuman primates infected with variola virus (Smallpox). Antimicrob. Agents Chemother. 57, 6246–6253. doi: 10.1128/AAC.00977-13 24100494 PMC3837858

[B92] Mullard A. (2018). Re-assessing the rule of 5, two decades on. Nat. Rev. Drug Discovery 17, 777–777. doi: 10.1038/nrd.2018.197 30374178

[B93] Niaz F. Tariq S. Ali S. M. Memon R. Nashwan A. J. Ullah I. (2022). Monkeypox Treatment: Is Tecovirimat the Answer? J. Infect. Public Health 15, 1298. doi: 10.1016/J.JIPH.2022.10.012 PMC9573899 36274371

[B94] Nguyen T. T. Viet M. H. Li M. S. (2014). Effects of water models on binding affinity: Evidence from all-atom simulation of binding of tamiflu to A/H5N1 neuraminidase. Sci. World J. doi: 10.1155/2014/536084 PMC3929574 24672329

[B95] Ntie-Kang F. Zofou D. Babiaka S. B. Meudom R. Scharfe M. Lifongo L. L.. (2013). AfroDb: A select highly potent and diverse natural product library from African medicinal plants. PloS One 8, e78085. doi: 10.1371/journal.pone.0078085 24205103 PMC3813505

[B96] Owens R. J. Anantharamaiah G. M. Kahlon J. B. Srinivas R. V. Compans R. W. Segrest J. P. (1990). Apolipoprotein A-I and its amphipathic helix peptide analogues inhibit human immunodeficiency virus-induced syncytium formation. J. Clin. Invest. 86, 1142–1150. doi: 10.1172/JCI114819 2170446 PMC296843

[B97] Owens L. E. Currie D. W. Kramarow E. A. Siddique S. Swanson M. Carter R. J.. (2023). JYNNEOS vaccination coverage among persons at risk for mpox — United states, may 22, 2022–January 31, 2023. MMWR Morb Mortal Wkly Rep. 72, 342–347. doi: 10.15585/MMWR.MM7213A4 36995962 PMC10078841

[B98] Pallio G. Broni E. Ashley C. Adams J. Manu H. Aikins E.. (2023). Cheminformatics-based study identifies potential ebola VP40 inhibitors. Int. J. Mol. Sci. 24, 6298. doi: 10.3390/IJMS24076298 37047270 PMC10094735

[B99] Parasuraman S. (2011). Prediction of activity spectra for substances. J. Pharmacol. Pharmacother. 2, 52–53. doi: 10.4103/0976-500X.77119 21701651 PMC3117574

[B100] Paredes A. Alzuru M. Mendez J. Rodríguez-Ortega M. (2003). Anti-sindbis activity of flavanones hesperetin and naringenin. Biol. Pharm. Bull. 26, 108–109. doi: 10.1248/bpb.26.108 12520185

[B101] Parker S. Buller R. M. A. (2013). review of experimental and natural infections of animals with monkeypox virus between 1958 and 2012. Future Virol. 8, 129–157. doi: 10.2217/fvl.12.130 23626656 PMC3635111

[B102] Pastula D. M. Copeland M. J. Hannan M. C. Rapaka S. Kitani T. Kleiner E.. (2022). Two cases of monkeypox-associated encephalomyelitis — Colorado and the district of Columbia, July–August 2022. MMWR Recomm Rep. 71, 1212–1215. doi: 10.15585/mmwr.mm7138e1 PMC9531567 36136957

[B103] Patil R. Das S. Stanley A. Yadav L. Sudhakar A. Varma A. K. (2010). Optimized Hydrophobic Interactions and Hydrogen Bonding at the Target-Ligand Interface Leads the Pathways of Drug-Designing. PLoS One 5, e12029. doi: 10.1371/journal.pone.0012029 20808434 PMC2922327

[B104] Preet G. Oluwabusola E. T. Milne B. F. Ebel R. Jaspars M. (2022). Computational repurposing of mitoxantrone-related structures against monkeypox virus: A molecular docking and 3D pharmacophore study. Int. J. Mol. Sci. 23, 14287. doi: 10.3390/ijms232214287 36430762 PMC9695275

[B105] Rabaan A. A. Abas A. H. Tallei T. E. Al-Zaher M. A. Al-Sheef N. M. Fatimawali. (2023). Monkeypox outbreak 2022: What we know so far and its potential drug targets and management strategies. J. Med. Virol. 95 (1), e28306. doi: 10.1002/jmv.28306 36372558

[B106] Ramírez D. Caballero J. (2018). Is it reliable to take the molecular docking top scoring position as the best solution without considering available structural data? Molecules 23, 1038. doi: 10.3390/molecules23051038 29710787 PMC6102569

[B107] Rappe A. K. Casewit C. J. Colwell K. S. Goddard W. A. Skiff W. M. (1992). UFF, a full periodic table force field for molecular mechanics and molecular dynamics simulations. J. Am. Chem. Soc. 114, 10024–10035. doi: 10.1021/ja00051a040

[B108] Realegeno S. Puschnik A. S. Kumar A. Goldsmith C. Burgado J. Sambhara S.. (2017). Monkeypox virus host factor screen using haploid cells identifies essential role of GARP complex in extracellular virus formation. J. Virol. 91 (11), e00011-17. doi: 10.1128/jvi.00011-17 28331092 PMC5432867

[B109] Reeves P. M. Bommarius B. Lebeis S. McNulty S. Christensen J. Swimm A.. (2005). Disabling poxvirus pathogenesis by inhibition of Abl-family tyrosine kinases. Nat. Med. 11, 731–739. doi: 10.1038/nm1265 15980865

[B110] Reeves P. M. Smith S. K. Olson V. A. Thorne S. H. Bornmann W. Damon I. K.. (2011). Variola and monkeypox viruses utilize conserved mechanisms of virion motility and release that depend on abl and src family tyrosine kinases. J. Virol. 85, 21–31. doi: 10.1128/jvi.01814-10 20962097 PMC3014172

[B111] Rose P. W. Prlić A. Altunkaya A. Bi C. Bradley A. R. Christie C. H.. (2017). The RCSB protein data bank: Integrative view of protein, gene and 3D structural information. Nucleic Acids Res. 45, D271–D281. doi: 10.1093/nar/gkw1000 27794042 PMC5210513

[B112] Sadeuh-Mba S. A. Yonga M. G. Els M. Batejat C. Eyangoh S. Caro V.. (2019). Monkeypox virus phylogenetic similarities between a human case detected in Cameroon in 2018 and the 2017-2018 outbreak in Nigeria. Infect Genet Evol. 69, 8–11. doi: 10.1016/j.meegid.2019.01.006 30634001 PMC9533929

[B113] Sander T. Freyss J. Von Korff M. Rufener C. (2015). DataWarrior: An open-source program for chemistry aware data visualization and analysis. J. Chem. Inf Model. 55, 460–473. doi: 10.1021/ci500588j 25558886

[B114] Sekiguchi J. Stivers J. T. Mildvan A. S. Shuman S. (1996). Mechanism of inhibition of vaccinia DNA topoisomerase by novobiocin and coumermycin. J. Biol. Chem. 271, 2313–2322. doi: 10.1074/jbc.271.4.2313 8567695

[B115] Sepehrinezhad A. Ashayeri Ahmadabad R. Sahab-Negah S. (2023). Monkeypox virus from neurological complications to neuroinvasive properties: current status and future perspectives. J. Neurol. 270, 101–108. doi: 10.1007/s00415-022-11339-w 35989372 PMC9393054

[B116] Sgobba M. Caporuscio F. Anighoro A. Portioli C. Rastelli G. (2012). Application of a post-docking procedure based on MM-PBSA and MM-GBSA on single and multiple protein conformations. Eur. J. Med. Chem. 58, 431–440. doi: 10.1016/j.ejmech.2012.10.024 23153814

[B117] Shawon J. Khan A. M. Shahriar I. Halim M. A. (2021). Improving the binding affinity and interaction of 5-Pentyl-2-Phenoxyphenol against Mycobacterium Enoyl ACP reductase by computational approach. Inf. Med. Unlocked 23, 100528. doi: 10.1016/j.imu.2021.100528

[B118] Sherwat A. Brooks J. T. Birnkrant D. Kim P. (2022). Tecovirimat and the treatment of monkeypox — Past, present, and future considerations. N Engl. J. Med. 387, 579–581. doi: 10.1056/NEJMp2210125 35921403

[B119] Singh I. P. Chopra A. K. Coppenhaver D. H. Ananatharamaiah G. M. Baron S. (1999). Lipoproteins account for part of the broad non-specific antiviral activity of human serum. Antiviral Res. 42 (3), 211–8. doi: 10.1016/s0166-3542(99)00032-7 10443533

[B120] Silva F. Perez da Graça J. Porto C. Martin do Prado R. Nunes E. Corrêa Marcelino-Guimarães E.. (2018). Untargeted metabolomics analysis by UHPLC-MS/MS of soybean plant in a compatible response to phakopsora pachyrhizi infection. Metabolitese 11, 179. doi: 10.3390/metabo11030179 PMC8003322 33808519

[B121] Sliva K. Schnierle B. (2007). From actually toxic to highly specific - Novel drugs against poxviruses. Virol. J. 4, 8. doi: 10.1186/1743-422X-4-8 17224068 PMC1781423

[B122] Srinivas R. V. Birkedal B. Owens R. J. Anantharamaiah G. M. Segrest J. P. Compans R. W. (1990). Antiviral effects of apolipoprotein A-I and its synthetic amphipathic peptide analogs. Virology 176, 48–57. doi: 10.1016/0042-6822(90)90229-K 2158697

[B123] Sterling T. Irwin J. J. (2015). ZINC 15 – ligand discovery for everyone. J. Chem. Inf Model. 55, 2324–2337. doi: 10.1021/acs.jcim.5b00559 26479676 PMC4658288

[B124] Takada N. Sanda T. Okamoto H. Yang J.-P. Asamitsu K. Sarol L.. (2002). RelA-associated inhibitor blocks transcription of human immunodeficiency virus type 1 by inhibiting NF-κB and sp1 actions. J. Virol. 76, 8019–8030. doi: 10.1128/jvi.76.16.8019-8030.2002 12134007 PMC155123

[B125] Tayyaba U. Sultan A. Khan F. Ahmed S. Ahmad I. (2022). Monkeypox: A review in Indian context. J. Pure Appl. Microbiol. 16, 3025–3035. doi: 10.22207/JPAM.16.SPL1.05

[B126] Tian W. Chen C. Lei X. Zhao J. Liang J. (2018). CASTp 3.0: Computed atlas of surface topography of proteins. Nucleic Acids Res. doi: 10.1093/nar/gky473 PMC6031066 29860391

[B127] Tomori O. Ogoina D. (2022). Monkeypox: The consequences of neglecting a disease, anywhere. Science 377, 1261–1263. doi: 10.1126/science.add3668 36107995

[B128] Trott O. Olson A. J. (2010). Software news and update AutoDock Vina: Improving the speed and accuracy of docking with a new scoring function, efficient optimization, and multithreading. J. Comput. Chem. doi: 10.1002/jcc.21334 PMC3041641 19499576

[B129] Ulubelen A. Öztürk M. (2006). Alkaloids and coumarins from ruta species. Nat. Prod Commun. 1, 851–857. doi: 10.1177/1934578x0600101006

[B130] Urmi U. L. Willcox M. D. P. Islam S. Kuppusamy R. Vijay A. K. (2023). Ocular signs and symptoms of monkeypox virus infection, and possible role of the eye in transmission of the virus. Contact Lens Anterior Eye 46, 101808. doi: 10.1016/j.clae.2022.101808 36585302 PMC9795335

[B131] Veber D. F. Johnson S. R. Cheng H. Smith B. R. Ward K. W. Kopple K. D. (2002). Molecular properties that influence the oral bioavailability of drug candidates. J. Med. Chem. 45, 2615–2623. doi: 10.1021/jm020017n 12036371

[B132] Wang J. Prinz R. A. Liu X. Xu X. (2020). *In vitro* and in *vivo* antiviral activity of gingerenone a on influenza a virus is mediated by targeting janus kinase 2. Viruses 12, 1141. doi: 10.3390/v12101141 33050000 PMC7650803

[B133] Warner B. M. Klassen L. Sloan A. Deschambault Y. Soule G. Banadyga L.. (2022). *In vitro* and in *vivo* efficacy of tecovirimat against a recently emerged 2022 monkeypox virus isolate. Sci. Transl. Med. 14, eade7646. doi: 10.1126/scitranslmed.ade7646 36318038

[B134] Wishart D. S. Feunang Y. D. Guo A. C. Lo E. J. Marcu A. Grant J. R.. (2018). DrugBank 5.0: A major update to the DrugBank database for 2018. Nucleic Acids Res. 46, D1074–D1082. doi: 10.1093/nar/gkx1037 29126136 PMC5753335

[B135] Wishart D. S. Knox C. Guo A. C. Cheng D. Shrivastava S. Tzur D.. (2008). DrugBank: a knowledgebase for drugs, drug actions and drug targets. Nucleic Acids Res. 36, D901–D906. doi: 10.1093/nar/gkm958 18048412 PMC2238889

[B136] Xiang Y. White A. (2022). Monkeypox virus emerges from the shadow of its more infamous cousin: family biology matters. Emerg. Microbes Infect. 11, 1768–1777. doi: 10.1080/22221751.2022.2095309 35751396 PMC9278444

[B137] Xin W. Huang H. Yu L. Shi H. Sheng Y. Wang T. T. Y. (2012). Three new flavanonol glycosides from leaves of engelhardtia roxburghiana, and their anti-inflammation, antiproliferative and antioxidant properties. Food Chem 132, 788–798. doi: 10.1016/j.foodchem.2011.11.038

[B138] Xu B. Wang L. González-Molleda L. Wang Y. Xu J. Yuan Y. (2014). Antiviral activity of (+)-rutamarin against kaposi’s sarcoma- associated herpesvirus by inhibition of the catalytic activity of human topoisomerase II. Antimicrob. Agents Chemother. 58, 563–573. doi: 10.1128/AAC.01259-13 24295975 PMC3910736

[B139] Yue L. Yi L. Fei T. MengWu T. Man L. LiQing W.. (2022). Human encephalitis complicated with ocular symptoms associated with pseudorabies virus infection: A case report. Front. Neurol. 13. doi: 10.3389/fneur.2022.878007 PMC9125146 35614923

[B140] Zardi E. M. Chello C. (2022). Human monkeypox—A global public health emergency. Int. J. Environ. Res. Public Health 19, 16781. doi: 10.3390/ijerph192416781 36554659 PMC9779584

[B141] Zhang W.-H. Wilcock D. Smith G. L. (2000). Vaccinia virus F12L protein is required for actin tail formation, normal plaque size, and virulence. J. Virol. 74, 11654–11662. doi: 10.1128/JVI.74.24.11654-11662.2000 11090164 PMC112447

[B142] Zhou Q. Yan L. Xu B. Wang X. Sun X. Han N.. (2021). Screening of the HBx transactivation domain interacting proteins and the function of interactor Pin1 in HBV replication. Sci. Rep. 11, 14176. doi: 10.1038/s41598-021-93584-z 34238995 PMC8266847

