# Supplementary material for: Identifying potential monkeypox virus inhibitors: an in silico study targeting the A42R protein
Source: Front Cell Infect Microbiol. 2024 Mar 4;14:1351737. doi: 10.3389/fcimb.2024.1351737 (PMC10945028; doi:10.3389/fcimb.2024.1351737)
Supplement: Supplementary file 1 [file DataSheet_1.docx]

**Supplementary Information**

**Identifying Potential Monkeypox Virus Inhibitors: An *In Silico* Study Targeting the A42R Protein**

Carolyn Ashley ^1^, Emmanuel Broni ^1^, ChaNyah M. Wood ^1,2^, Tunmise Okuneye ^1,3^, Mary-Pearl T. Ojukwu ^1,2,4^, Qunfeng Dong ^1,5^, Carla Gallagher ^2^, and Whelton A. Miller III ^1,6^*

^1^ Department of Medicine, Loyola University Medical Center, Loyola University Chicago, Maywood, IL 60153, USA; [cashley1@luc.edu](mailto:cashley1@luc.edu) (C.A.); [ebroni@luc.edu](mailto:ebroni@luc.edu) (E.B.); [qdong@luc.edu](mailto:qdong@luc.edu) (Q.D.)

^2^ Department of Chemistry and Physics, Lincoln University, PA 19352, USA; [chanyahmwood@gmail.com](mailto:chanyahmwood@gmail.com) (C.W.); [M.ojukwu@ufl.edu](mailto:M.ojukwu@ufl.edu) (M.T.O.); [cgallagher@lincoln.edu](mailto:cgallagher@lincoln.edu) (C.G.)

^3^ Department of Biology, Lincoln University, PA 19352, USA; [tunmise.okuneye@lions.lincoln.edu](mailto:tunmise.okuneye@lions.lincoln.edu) (T.O.)

^4^ College of Pharmacy, University of Florida, Orlando, FL 32827, USA;

^5^ Center for Biomedical Informatics, Stritch School of Medicine, Loyola University Chicago, Maywood, IL 60153, USA;

^6^ Department of Molecular Pharmacology & Neuroscience, Loyola University Medical Center, Loyola University Chicago, Maywood, IL 60153, USA;

***** Correspondence: [wmiller6@luc.edu](mailto:wmiller6@luc.edu)

**Content**

Figure S1: Potential energies comparison for A42R energy minimization using OPLS/AA and CHARMM36 force fields.

Figure S2: Protein-ligand interaction plots (PLIP) of top compounds A) PubChem CID: 11371862, B) ZINC000001632866, C) ZINC000013378519, D) ZINC000000086470, and E) ZINC000095486204, and F) known inhibitor, tecovirimat. Black circles are carbon, red circles are oxygen, blue circles are nitrogen, and yellow circles are sulfur. Residue names in green interact in hydrogen bonding, dashed green lines are the hydrogen bond representation. Black residues are interacting by hydrophobic bonds corresponding to red markings on the ligands.

Figure S3: Hydrogen bond analysis of A42R in complex with tecovirimat and top compounds ZINC000000899909, ZINC000001632866, ZINC000015151344, ZINC000013378519, ZINC000000086470, ZINC000095486204, and PC11371962.

Figure S4: Molecular mechanics Poisson-Boltzmann surface area (MM/PBSA) plots showing the per residue energy contributions for A) A42R-ZINC000001632866, B) A42R-ZINC000015151344, C) A42R-ZINC000000086470, D) A42R-ZINC000095486204, E) A42R-PC11371962, and F) A42R-tecovirimat complexes. Critical residue fluctuations (>5 and < -5 kJ/mol) are colored red.

Table S1: Docking scores from Autodock Vina and OSIRIS DataWarrior toxicity predictions of shortlisted compounds from TCM, AfroDB, and PubChem that passed ADME predictions. MPXV inhibitor, tecovirimat is included in the table. Table cells are labeled “None” in green, “Low” in yellow, and “High” in red for the toxicity prediction from DataWarrior 5.5.0.

Table S2: Summary of important biological activity predictions for seven potential lead compounds A) ZINC000000899909, B) ZINC000001632866, C) ZINC000015151344, D) ZINC000013378519, E) ZINC000000086470, F) ZINC000095486204, and G) PC11371962 from Prediction of Activity Spectra of Substances (PASS). Pa is potential for activity and Pi is potential of inactivity.

**Supplementary Figures**


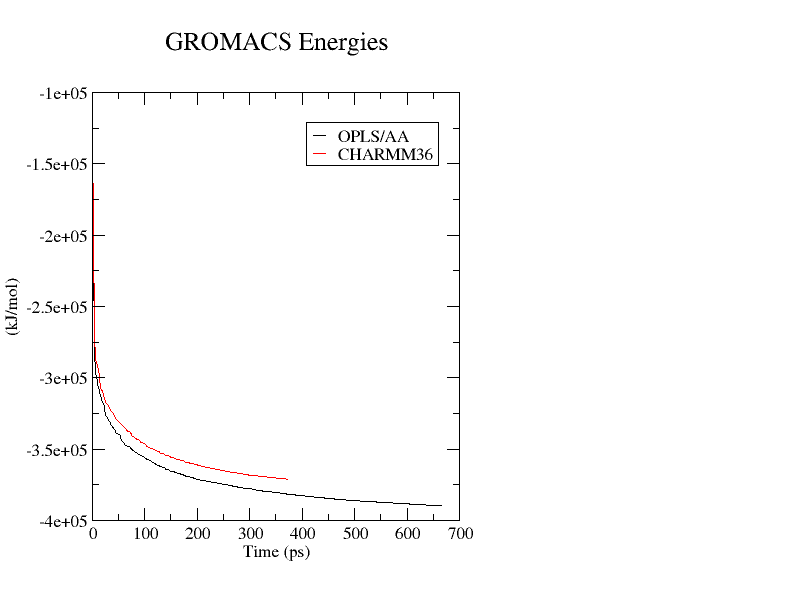


Figure S1: Potential energies comparison for A42R energy minimization using OPLS/AA and CHARMM36 force fields.

A)


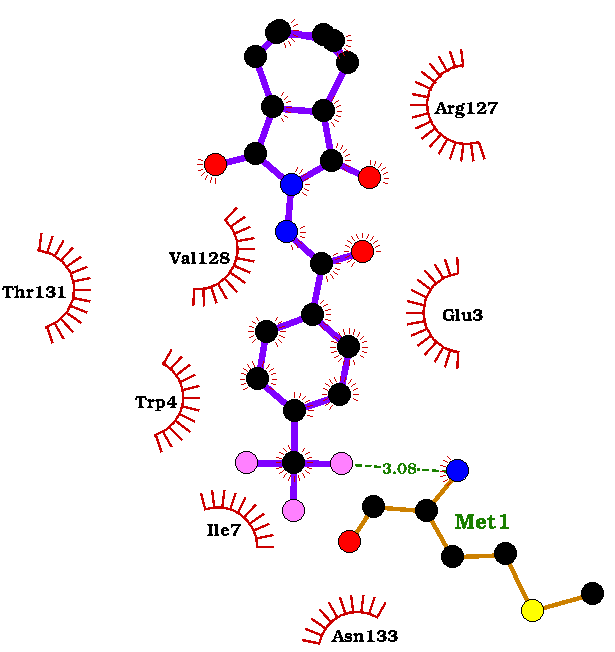


B)


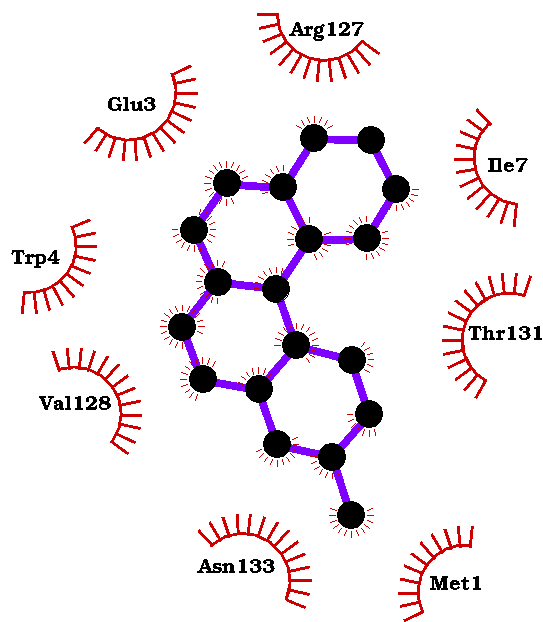


C)


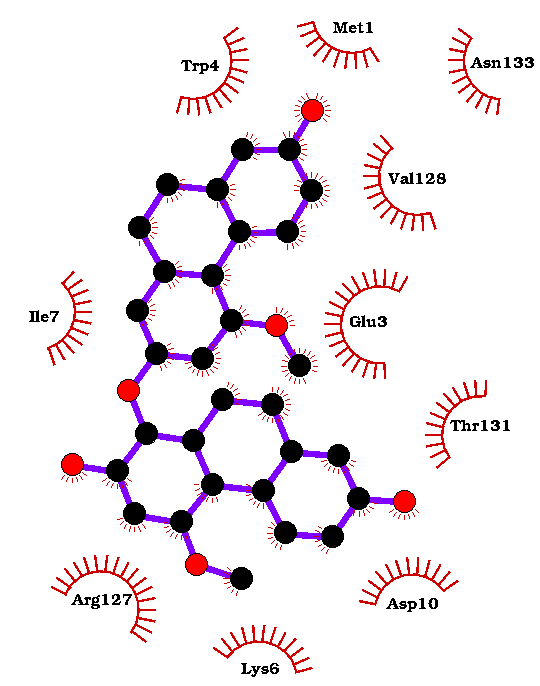


D)


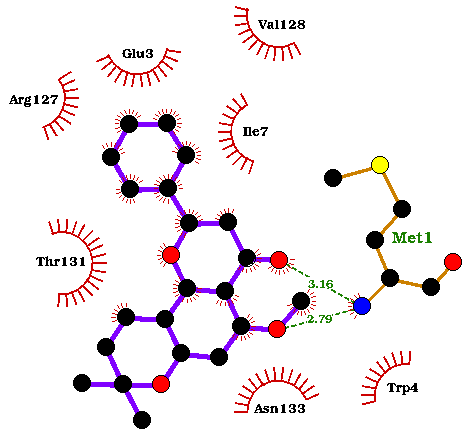


E)


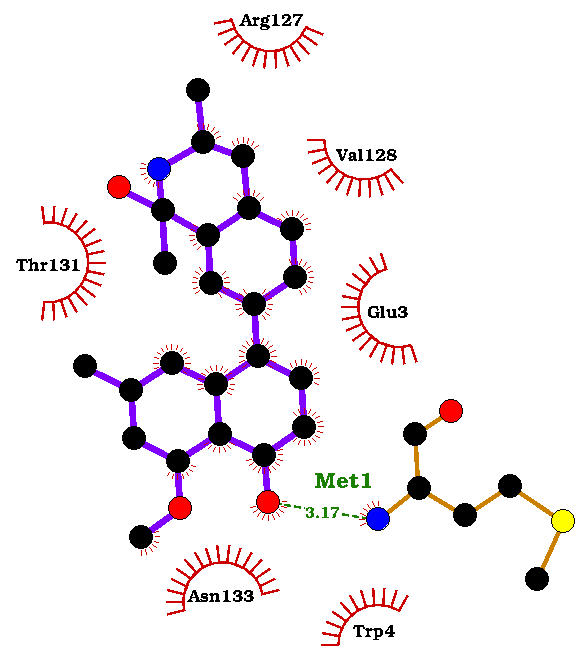


F)


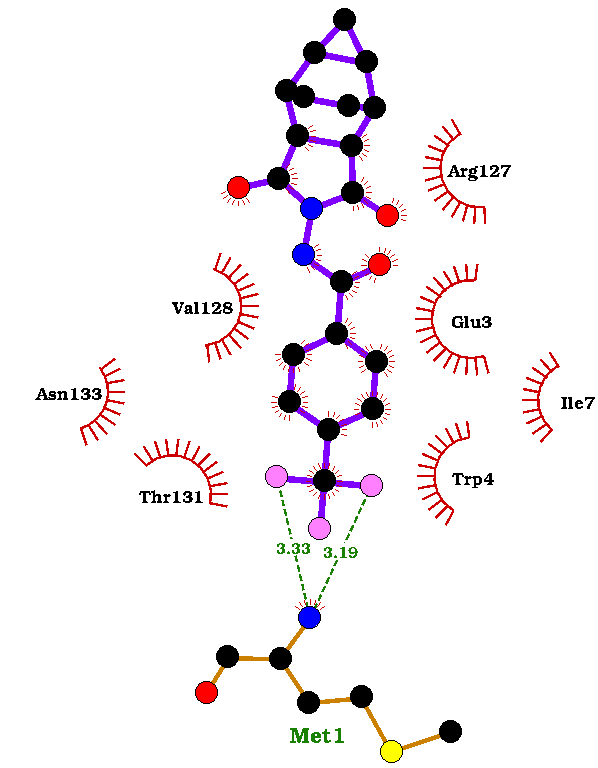


Figure S2: Protein-ligand interaction plots (PLIP) of top compounds A) PubChem CID: 11371862, B) ZINC000001632866, C) ZINC000013378519, D) ZINC000000086470, and E) ZINC000095486204, and F) known inhibitor, tecovirimat. Black circles are carbon, red circles are oxygen, blue circles are nitrogen, and yellow circles are sulfur. Residue names in green interact in hydrogen bonding, dashed green lines are the hydrogen bond representation. Black residues are interacting by hydrophobic bonds corresponding to red markings on the ligands.


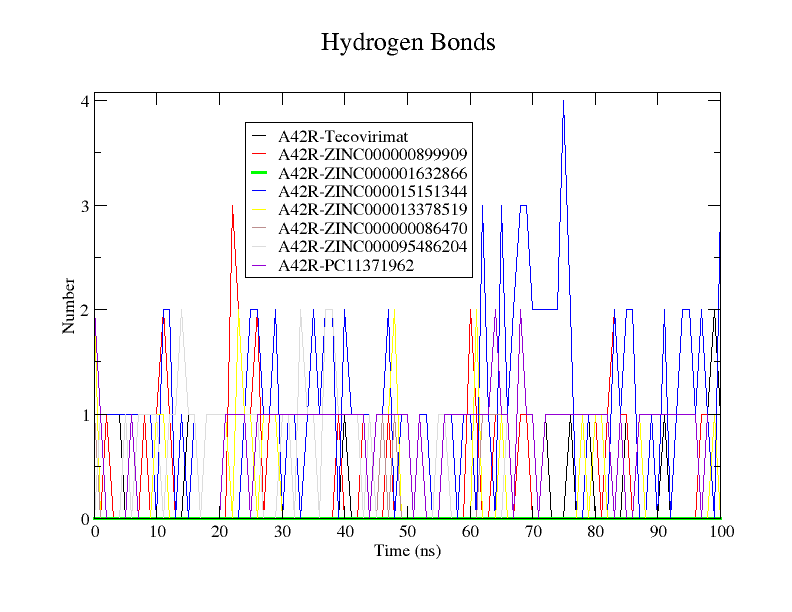


Figure S3: Hydrogen bond analysis of A42R in complex with tecovirimat and top compounds ZINC000000899909, ZINC000001632866, ZINC000015151344, ZINC000013378519, ZINC000000086470, ZINC000095486204, and PC11371962.

A)


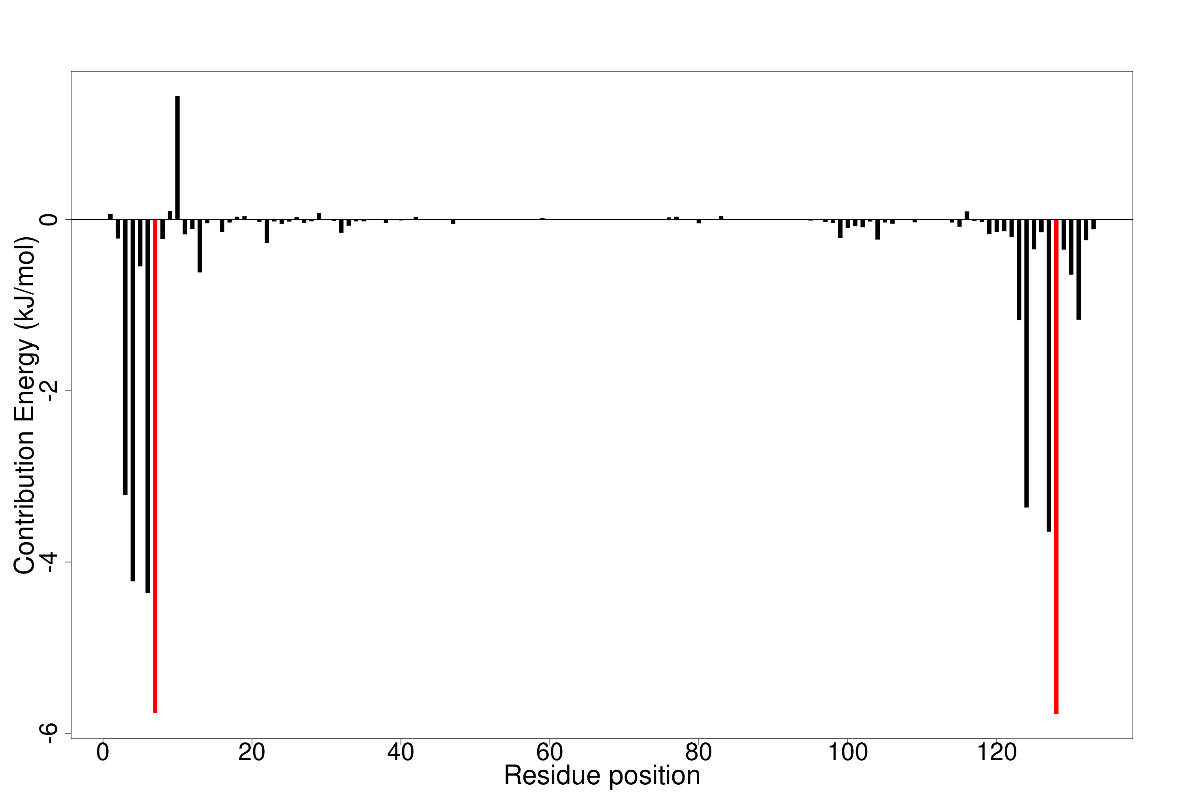


B)


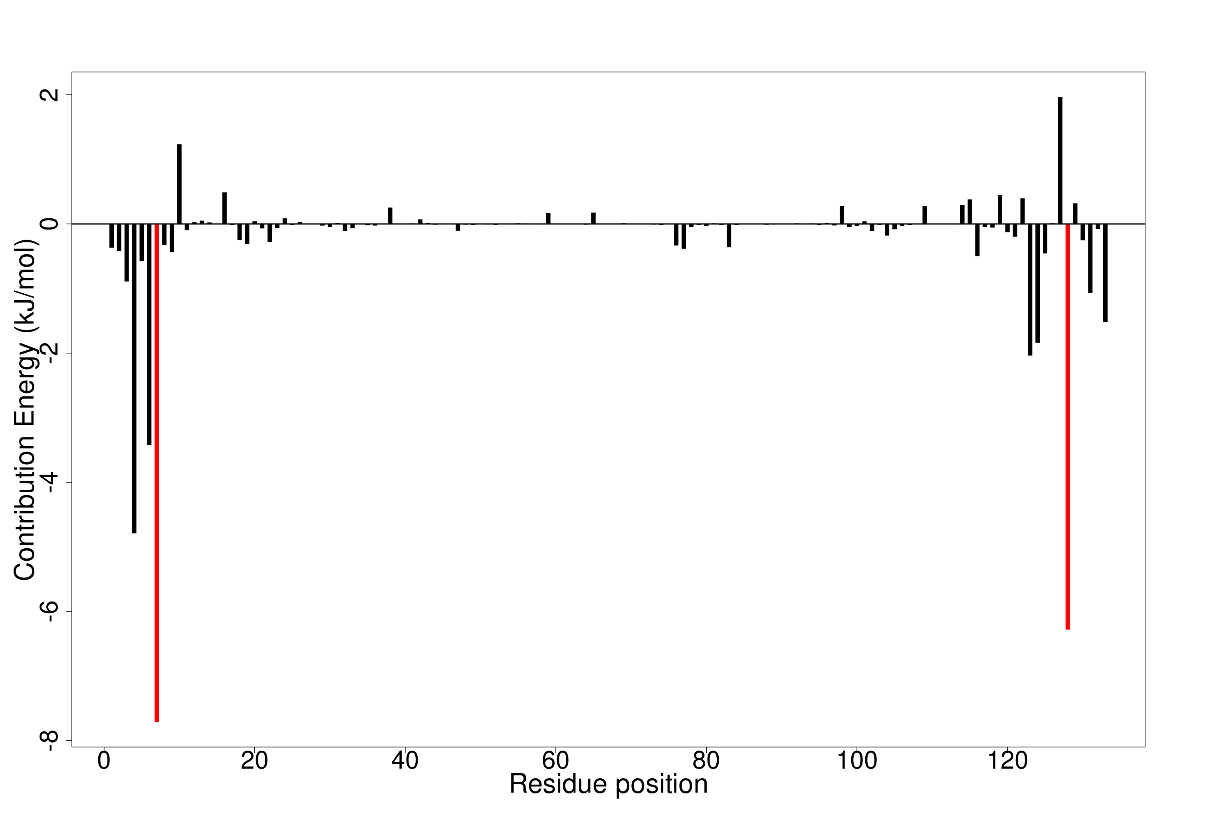


C)


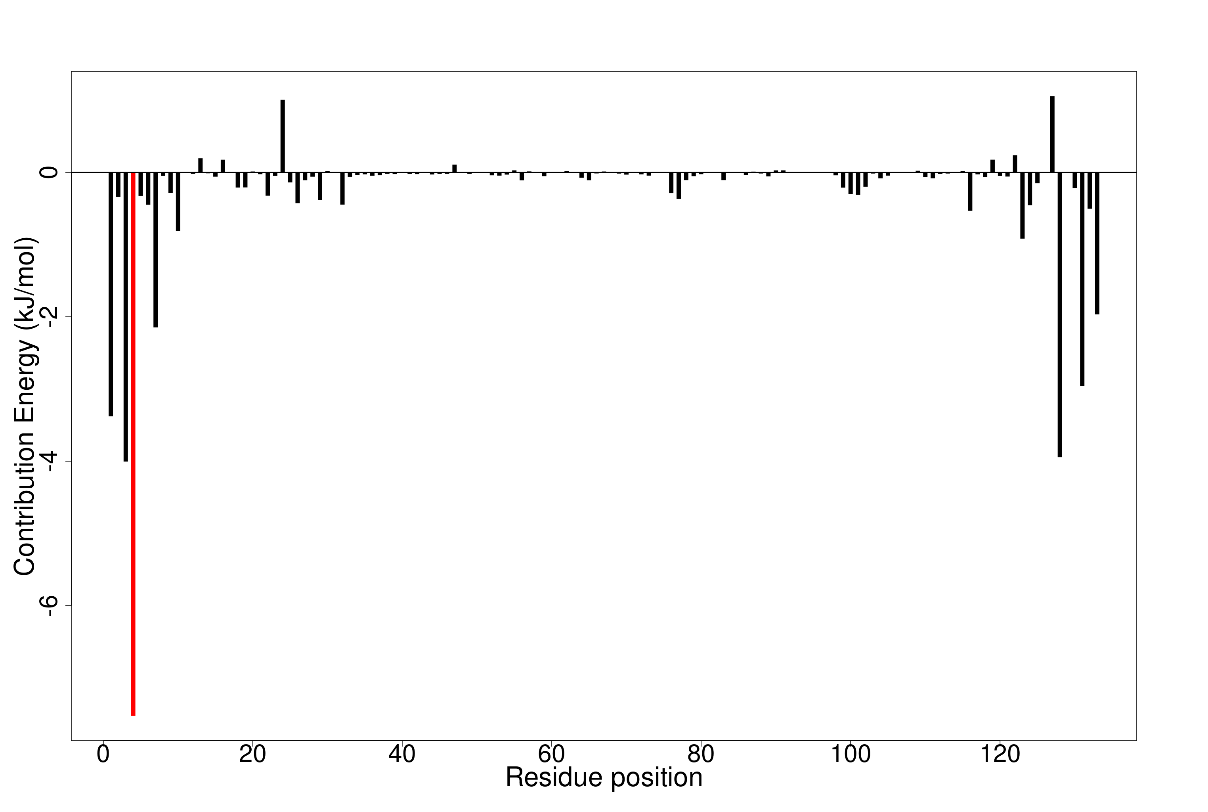


D)


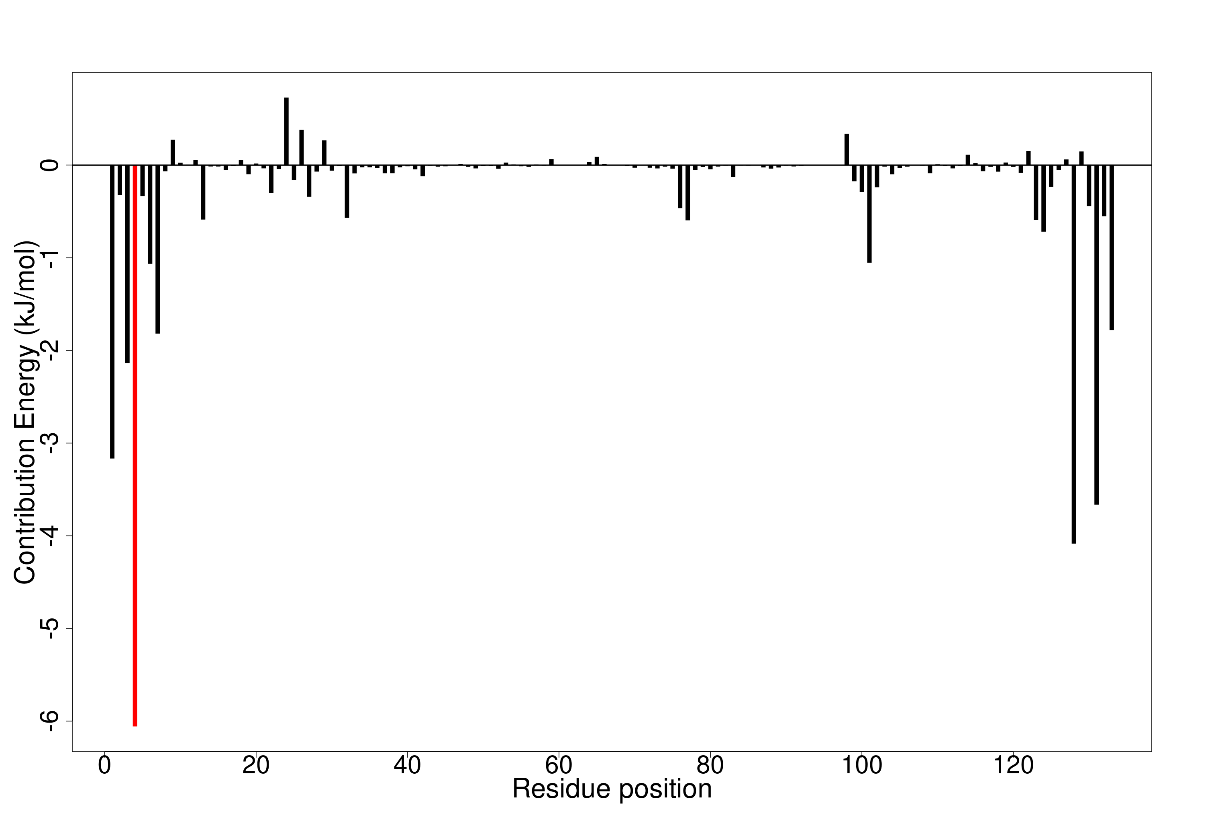


E)


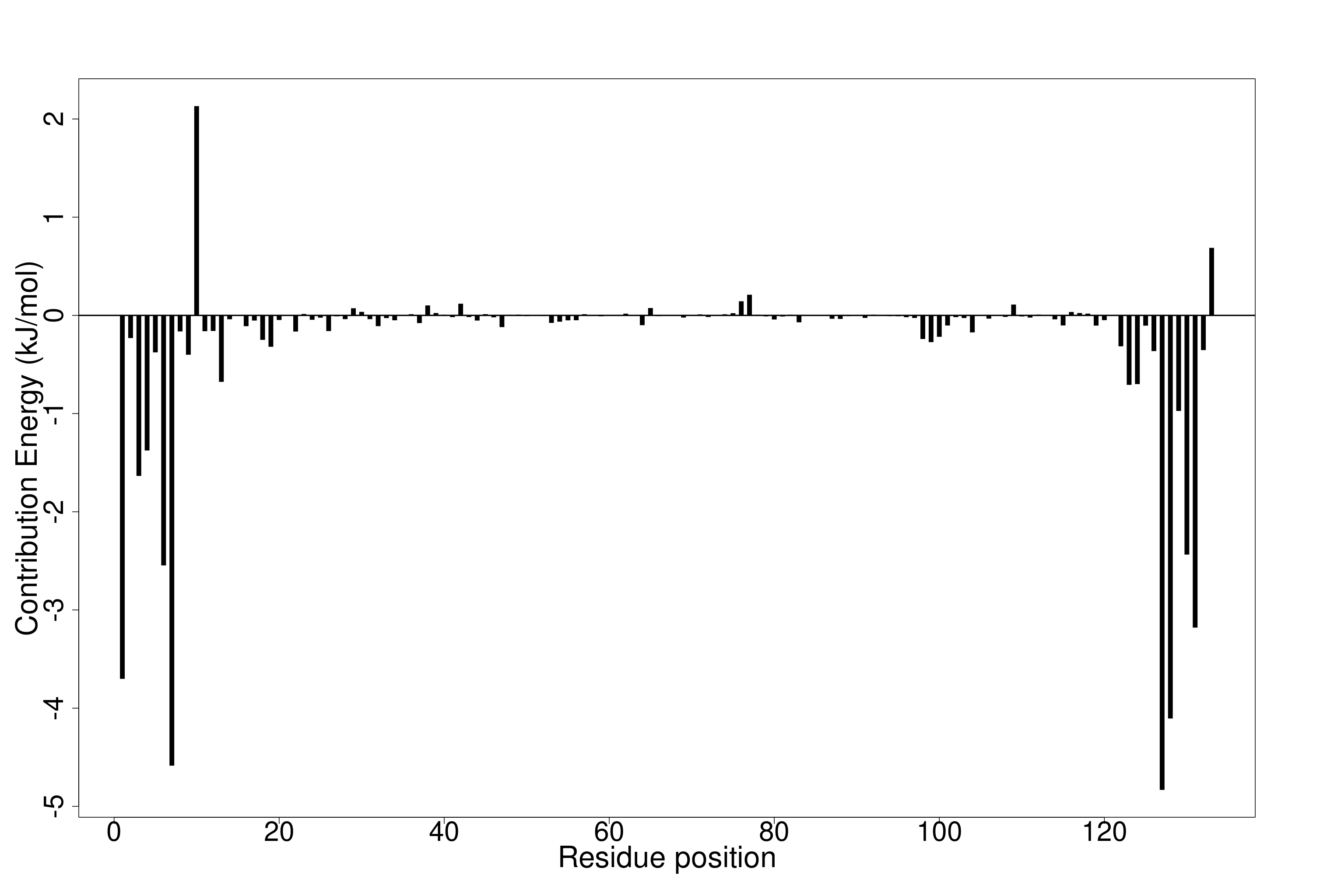


F)


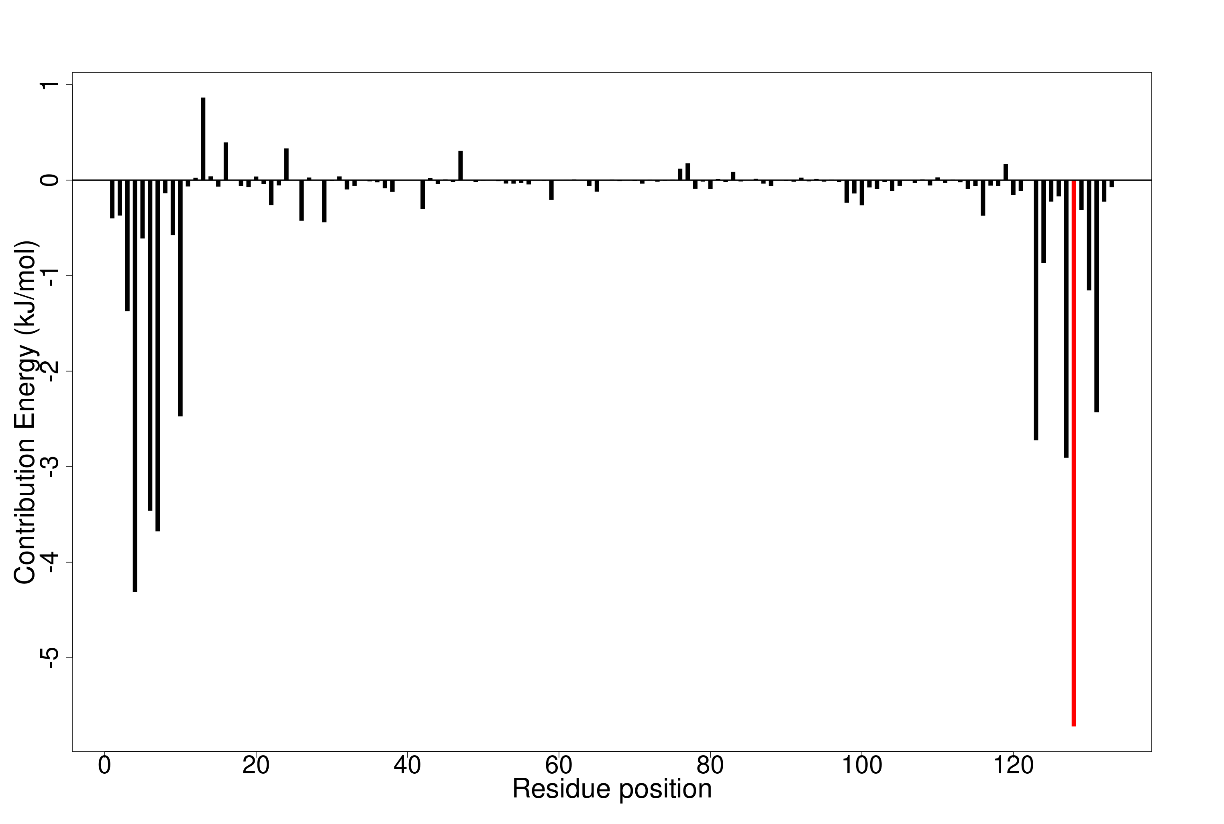


Figure S4: Molecular mechanics Poisson-Boltzmann surface area (MM/PBSA) plots showing the per residue energy contributions for A) A42R-ZINC000001632866, B) A42R-ZINC000015151344, C) A42R-ZINC000000086470, D) A42R-ZINC000095486204, E) A42R-PC11371962, and F) A42R-tecovirimat complexes. Critical residue fluctuations (>5 and < -5 kJ/mol) are colored red.

**Supplementary Tables**

Table S1: Docking scores from Autodock Vina and OSIRIS DataWarrior toxicity predictions of shortlisted compounds from TCM, AfroDB, and PubChem that passed ADME predictions. MPXV inhibitor, tecovirimat is included in the table. Table cells are labeled “None” in green, “Low” in yellow, and “High” in red for the toxicity prediction from DataWarrior 5.5.0.

| **Compound** | **Binding Energy (kcal/mol)** | **Mutagenic** | **Tumorigenic** | **Reproductive Effective** | **Irritant** |
| --- | --- | --- | --- | --- | --- |
| ZINC000095486204 | -8.3 | none | high | low | none |
| ZINC000013378519 | -8.1 | none | none | none | none |
| ZINC000001632866 | -8 | low | high | none | none |
| ZINC000015151344 | -7.9 | none | none | none | none |
| ZINC000000899909 | -7.8 | none | none | high | none |
| ZINC000095909830 | -7.8 | none | none | none | none |
| ZINC000095913878 | -7.8 | none | none | none | none |
| ZINC000000689683 | -7.7 | none | none | none | none |
| ZINC000000897930 | -7.7 | none | none | none | none |
| ZINC000013375730 | -7.7 | none | none | high | none |
| ZINC000028702248 | -7.7 | low | high | high | high |
| ZINC000059589174 | -7.7 | none | none | high | none |
| ZINC000085594093 | -7.6 | low | none | high | high |
| ZINC000000086470 | -7.6 | none | none | high | none |
| ZINC000000134782 | -7.4 | none | none | none | none |
| ZINC000031852149 | -7.3 | none | high | none | high |
| ZINC000095485910 | -7.3 | high | high | none | none |
| Pub11371962 | -7.2 | none | none | high | none |
| ZINC000048998695 | -7.2 | none | none | none | none |
| ZINC000095486327 | -7.1 | none | high | none | none |
| ZINC000014557836 | -7 | none | none | none | none |
| ZINC000038658035 | -7 | none | none | none | none |
| Pub11360575 | -7 | none | none | low | none |
| Pub129016384 | -7 | none | none | high | none |
| Tecovirimat | -6.7 | none | none | high | none |

Table S2: Summary of important biological activity predictions for seven potential lead compounds A) ZINC000000899909, B) ZINC000001632866, C) ZINC000015151344, D) ZINC000013378519, E) ZINC000000086470, F) ZINC000095486204, and G) PC11371962 from Prediction of Activity Spectra of Substances (PASS). Pa is potential for activity and Pi is potential of inactivity.

**A**.

| **ZINC000000899909** | | |
| --- | --- | --- |
| Activity | Pa | Pi |
| RELA expression inhibitor | 0,623 | 0,003 |
| JAK2 expression inhibitor | 0,527 | 0,047 |
| Anticarcinogenic | 0,470 | 0,022 |
| HCV IRES inhibitor | 0,437 | 0,017 |
| APOA1 expression enhancer | 0,443 | 0,047 |
| Pin1 inhibitor | 0,361 | 0,112 |
| Topoisomerase I inhibitor | 0,272 | 0,018 |
| Topoisomerase II inhibitor | 0,147 | 0,035 |
| RNA directed DNA polymerase inhibitor | 0,275 | 0,038 |
| DNA directed RNA polymerase inhibitor | 0,209 | 0,033 |
| Antiviral (Herpes) | 0,438 | 0,021 |
| Antiviral (Rhinovirus) | 0,383 | 0,111 |
| Antiviral (Hepatitis B) | 0,212 | 0,075 |
| Viral entry inhibitor | 0,217 | 0,098 |
| Antiviral (HIV) | 0,132 | 0,090 |
| Antiviral | 0,164 | 0,142 |
| HIV-2 reverse transcriptase inhibitor | 0,217 | 0,027 |
| HIV-1 integrase inhibitor | 0,082 | 0,065 |

**B**.

| **ZINC000001632866** | | |
| --- | --- | --- |
| Activity | Pa | Pi |
| JAK2 expression inhibitor | 0,860 | 0,004 |
| Vasoprotector | 0,748 | 0,008 |
| Lipoprotein lipase inhibitor | 0,444 | 0,060 |
| RELA expression inhibitor | 0,493 | 0,012 |
| Pin1 inhibitor | 0,661 | 0,011 |
| Antiviral (Picornavirus) | 0,459 | 0,071 |
| Antiviral (Adenovirus) | 0,387 | 0,035 |
| Antiviral (CMV) | 0,299 | 0,030 |
| Antiviral (Poxvirus) | 0,315 | 0,052 |
| Viral entry inhibitor | 0,267 | 0,019 |
| Antiviral (Influenza) | 0,309 | 0,083 |
| Antiviral (Herpes) | 0,303 | 0,089 |
| Antiviral (Parainfluenza) | 0,059 | 0,015 |
| Antiviral (Rhinovirus) | 0,295 | 0,265 |
| HIV-2 reverse transcriptase inhibitor | 0,320 | 0,007 |
| HIV-1 integrase (Overall Integration) inhibitor | 0,205 | 0,013 |
| HIV fusion inhibitor | 0,019 | 0,003 |
| HCV IRES inhibitor | 0,292 | 0,096 |
| DNA directed DNA polymerase inhibitor | 0,132 | 0,052 |
| DNA polymerase I inhibitor | 0,243 | 0,167 |
| RNA-directed RNA polymerase inhibitor | 0,446 | 0,026 |

**C.**

| **ZINC000015151344** | | |
| --- | --- | --- |
| Activity | Pa | Pi |
| JAK2 expression inhibitor | 0,796 | 0,008 |
| Pin1 inhibitor | 0,636 | 0,013 |
| Vasoprotector | 0,607 | 0,019 |
| RELA expression inhibitor | 0,468 | 0,016 |
| RNA-directed RNA polymerase inhibitor | 0,414 | 0,043 |
| APOA1 expression enhancer | 0,420 | 0,059 |
| Antiviral (Adenovirus) | 0,381 | 0,037 |
| Antiviral (Influenza) | 0,388 | 0,050 |
| Antiviral (Picornavirus) | 0,386 | 0,121 |
| Antiviral (Herpes) | 0,322 | 0,077 |
| HCV IRES inhibitor | 0,315 | 0,072 |
| Antiviral (CMV) | 0,228 | 0,115 |
| Viral entry inhibitor | 0,214 | 0,109 |
| Antiviral (Poxvirus) | 0,215 | 0,136 |
| Antiviral (Hepatitis B) | 0,183 | 0,110 |
| Antiviral (HIV) | 0,133 | 0,088 |
| Antiviral (Rhinovirus) | 0,296 | 0,262 |
| Antiviral | 0,161 | 0,146 |
| HIV-2 reverse transcriptase inhibitor | 0,248 | 0,018 |
| HIV-1 integrase (Strand Transfer) inhibitor | 0,137 | 0,016 |
| HIV-1 integrase (3'-Processing) inhibitor | 0,134 | 0,017 |
| HIV-1 integrase inhibitor | 0,116 | 0,025 |
| HIV-1 integrase (Overall Integration) inhibitor | 0,105 | 0,055 |

**D.**

| **ZINC000013378519** | | |
| --- | --- | --- |
| Activity | Pa | Pi |
| JAK2 expression inhibitor | 0,902 | 0,003 |
| Pin1 inhibitor | 0,575 | 0,023 |
| Vasoprotector | 0,544 | 0,029 |
| Anticarcinogenic | 0,355 | 0,040 |
| Topoisomerase I inhibitor | 0,175 | 0,043 |
| RNA directed DNA polymerase inhibitor | 0,221 | 0,069 |
| DNA polymerase I inhibitor | 0,247 | 0,157 |
| RELA expression inhibitor | 0,544 | 0,006 |
| Topoisomerase II inhibitor | 0,146 | 0,035 |
| Antiviral (Herpes) | 0,316 | 0,080 |
| Viral entry inhibitor | 0,231 | 0,063 |
| Antiviral (Influenza) | 0,233 | 0,152 |
| HIV-1 integrase inhibitor | 0,110 | 0,030 |
| Antiviral (Hepatitis B) | 0,176 | 0,122 |
| HIV-1 integrase (Overall Integration) inhibitor | 0,146 | 0,028 |
| HIV-2 reverse transcriptase inhibitor | 0,156 | 0,060 |
| HIV-1 integrase (3'-Processing) inhibitor | 0,099 | 0,026 |
| HIV-1 integrase (Strand Transfer) inhibitor | 0,084 | 0,033 |

**E.**

| **ZINC000000086470** | | |
| --- | --- | --- |
| Activity | Pa | Pi |
| RELA expression inhibitor | 0,647 | 0,003 |
| Antiviral (Rhinovirus) | 0,568 | 0,009 |
| Anticarcinogenic | 0,553 | 0,015 |
| Antiviral (Herpes) | 0,452 | 0,018 |
| RNA directed DNA polymerase inhibitor | 0,421 | 0,016 |
| JAK2 expression inhibitor | 0,398 | 0,086 |
| Viral entry inhibitor | 0,297 | 0,007 |
| Antiviral (Influenza) | 0,343 | 0,067 |
| Antiviral (Hepatitis B) | 0,308 | 0,031 |
| Topoisomerase I inhibitor | 0,285 | 0,016 |
| Pin1 inhibitor | 0,360 | 0,113 |
| DNA directed RNA polymerase inhibitor | 0,235 | 0,027 |
| Vasoprotector | 0,319 | 0,137 |
| Topoisomerase II inhibitor | 0,123 | 0,046 |
| Antiviral | 0,158 | 0,152 |

**F.**

| **ZINC000095486204** | | |
| --- | --- | --- |
| Activity | Pa | Pi |
| JAK2 expression inhibitor | 0,620 | 0,029 |
| DNA polymerase I inhibitor | 0,553 | 0,005 |
| Pin1 inhibitor | 0,490 | 0,045 |
| RELA expression inhibitor | 0,370 | 0,047 |
| APOA1 expression enhancer | 0,362 | 0,104 |
| RNA directed DNA polymerase inhibitor | 0,264 | 0,043 |
| Vasoprotector | 0,326 | 0,129 |
| Viral entry inhibitor | 0,228 | 0,070 |
| DNA directed DNA polymerase inhibitor | 0,141 | 0,043 |
| Cancer associated disorders treatment | 0,257 | 0,162 |
| Anticarcinogenic | 0,190 | 0,132 |
| Topoisomerase I inhibitor | 0,113 | 0,084 |

**G.**

| **PC11371962** | | |
| --- | --- | --- |
| Activity | Pa | Pi |
| RNA-directed RNA polymerase inhibitor | 0,393 | 0,059 |
| DNA polymerase I inhibitor | 0,344 | 0,034 |
| Topoisomerase I inhibitor | 0,280 | 0,017 |
| Vasoprotector | 0,319 | 0,137 |
| 3C-like protease (Human coronavirus) inhibitor | 0,247 | 0,080 |
| Antiviral (Hepatitis) | 0,146 | 0,037 |
| JAK2 expression inhibitor | 0,263 | 0,164 |
| Pin1 inhibitor | 0,269 | 0,201 |
| RELA expression inhibitor | 0,222 | 0,199 |
